# Molecular identity of axonal sodium channels in human cortical pyramidal cells

**DOI:** 10.3389/fncel.2014.00297

**Published:** 2014-09-23

**Authors:** Cuiping Tian, Kaiyan Wang, Wei Ke, Hui Guo, Yousheng Shu

**Affiliations:** ^1^Institute of Neuroscience and State Key Laboratory of Neuroscience, Shanghai Institutes for Biological Sciences, Chinese Academy of Sciences, and University of Chinese Academy of SciencesShanghai, China; ^2^Department of Neurology, Huashan Hospital, Fudan UniversityShanghai, China; ^3^State Key Laboratory of Cognitive Neuroscience and Learning and International Data Group/McGovern Institute for Brain Research, School of Brain and Cognitive Sciences, Beijing Normal UniversityBeijing, China; ^4^Center for Collaboration and Innovation in Brain and Learning Sciences, Beijing Normal UniversityBeijing, China; ^5^Department of Neurosurgery, Shanghai Quyang Hospital, Tongji UniversityShanghai, China

**Keywords:** axon initial segment, human cortex, node of Ranvier, sodium channel subtype, parvalbumin, chandelier cell, pyramidal cell

## Abstract

Studies in rodents revealed that selective accumulation of Na^+^ channel subtypes at the axon initial segment (AIS) determines action potential (AP) initiation and backpropagation in cortical pyramidal cells (PCs); however, in human cortex, the molecular identity of Na^+^ channels distributed at PC axons, including the AIS and the nodes of Ranvier, remains unclear. We performed immunostaining experiments in human cortical tissues removed surgically to cure brain diseases. We found strong immunosignals of Na^+^ channels and two channel subtypes, Na_V_1.2 and Na_V_1.6, at the AIS of human cortical PCs. Although both channel subtypes were expressed along the entire AIS, the peak immunosignals of Na_V_1.2 and Na_V_1.6 were found at proximal and distal AIS regions, respectively. Surprisingly, in addition to the presence of Na_V_1.6 at the nodes of Ranvier, Na_V_1.2 was also found in a subpopulation of nodes in the adult human cortex, different from the absence of Na_V_1.2 in myelinated axons in rodents. Na_V_1.1 immunosignals were not detected at either the AIS or the nodes of Ranvier of PCs; however, they were expressed at interneuron axons with different distribution patterns. Further experiments revealed that parvalbumin-positive GABAergic axon cartridges selectively innervated distal AIS regions with relatively high immunosignals of Na_V_1.6 but not the proximal Na_V_1.2-enriched compartments, suggesting an important role of axo-axonic cells in regulating AP initiation in human PCs. Together, our results show that both Na_V_1.2 and Na_V_1.6 (but not Na_V_1.1) channel subtypes are expressed at the AIS and the nodes of Ranvier in adult human cortical PCs, suggesting that these channel subtypes control neuronal excitability and signal conduction in PC axons.

## INTRODUCTION

Neurons in the mammalian central nervous system (CNS) generate their main output signal, the action potential (AP), to encode information and communicate with other neurons. It is therefore essential to understand the organization of cellular compartments responsible for AP initiation and conduction. Although APs could be initiated independently at different subcellular compartments, including those in the dendrite and the axon (Andersen, 1960; Regehr et al., 1993; Mainen et al., 1995; Colbert and Johnston, 1996; Stuart et al., 1997a,b; Clark et al., 2005; Milojkovic et al., 2005; Meeks and Mennerick, 2007; Foust et al., 2010), early studies in spinal motor neurons (Coombs et al., 1957; Fatt, 1957; Fuortes et al., 1957; Eccles, 1964) and relatively recent work in cortical pyramidal cell (PC) revealed that the AIS is normally the AP initiation site (Mainen et al., 1995; Palmer and Stuart, 2006; Shu et al., 2007; Popovic et al., 2011; Kole and Stuart, 2012).

Activation of voltage-gated Na^+^ channels is responsible for the generation of APs. Theoretical studies suggested that a high density of Na^+^ channels at the AIS is required for AP initiation at the axon (Moore et al., 1983; Mainen et al., 1995; Rapp et al., 1996). Early immunostaining work in retinal ganglion cells revealed that Na^+^ channels concentrate at the axon hillock and the AIS (Wollner and Catterall, 1986); consistently, other studies in different cell types demonstrate that the Na^+^ channel density is high at the AIS (Boiko et al., 2003; Meeks and Mennerick, 2007; Van Wart et al., 2007; Lorincz and Nusser, 2008). In human cortical PCs, strong immunosignals of Na^+^ channels could be observed along the entire AIS, whereas no detectable signal was found at the soma and dendrites (Inda et al., 2006), suggesting much higher channel density at the AIS than that in the somatodendritic compartments. Immunostaining studies in rodents with antibodies of different Na^+^ channel subtypes revealed that channel subtypes may be selectively targeted to different regions of the AIS (Boiko et al., 2003; Van Wart et al., 2007; Duflocq et al., 2008) and could be subject to change in diseased states (Craner et al., 2003, 2004; Waxman, 2006). In rodent cortical PCs (Hu et al., 2009), channel subtype Na_V_1.2 and Na_V_1.6 accumulate at the proximal and distal AIS regions, respectively. Because channel subtypes differ in voltage-dependent properties (Rush et al., 2005), they may play distinct roles in AP initiation and propagation (Royeck et al., 2008; Hu et al., 2009). In this study, we sought to investigate the distribution pattern of different Na^+^ channel subtypes at the AIS of neurons in the human cortex.

After initiation APs can propagate othodromically along the axon and trigger neurotransmitter release at axon terminals. AP conduction also relies on the expression of Na^+^ channels along the axon. At early developmental stages of the CNS, unmyelinated axons mainly express Na_V_1.2 channels, which will be replaced by Na_V_1.6 channels as myelination proceeds (Boiko et al., 2001; Kaplan et al., 2001). In mature myelinated axons, Na_V_1.6 is the main subtype distributed at the nodes of Ranvier and plays critical roles in mediating saltatory AP conduction (Caldwell et al., 2000; Waxman, 2006). In this study, we also sought to reveal the molecular identity of Na^+^ channels distributed at the nodes of Ranvier in the adult human neocortex.

The AIS of PCs receives synaptic inputs from axo-axonic chandelier cells, a subtype of interneuron that expresses calcium binding protein parvalbumin (PV) and shows a non-adapting fast-spiking firing pattern (Somogyi, 1977; Fairen and Valverde, 1980; Somogyi et al., 1985; DeFelipe et al., 1989; Kawaguchi, 1995; Gonzalez-Burgos et al., 2005). It has been believed that this type of interneuron could inhibit postsynaptic PCs (Klausberger et al., 2003; Zhu et al., 2004; Tukker et al., 2007; Glickfeld et al., 2009); however, some other studies also revealed that chandelier cells could excite PCs (Szabadics et al., 2006; Khirug et al., 2008; Molnar et al., 2008), due to a high intracellular Cl^-^ concentration at the AIS of postsynaptic PCs. Whatever effect these cells produce, their strategic target location (i.e., the AIS) suggests that they may exert powerful control over AP initiation. Indeed, previous study in human cortical PCs indicates that axon terminals of chandelier cells preferentially target the distal regions of the AIS (Inda et al., 2006), corresponding to the AP initiation zone. Small fluctuations of the membrane potential or changes in membrane conductance at the distal AIS, due to the arrival of synaptic inputs from chandelier cells, may efficiently change the impact of local Na^+^ channels and thus regulate AP initiation. Because Na_V_1.2 and Na_V_1.6 channels accumulate at different AIS regions, it is of interest to examine the spatial relationship between specific channel subtypes and the axon terminals of chandelier cells.

With weak tissue fixation, our immunostaining experiments demonstrate strong immunosignals of Na^+^ channels at the AIS of human cortical PCs with a segregated distribution pattern of proximal-Na_V_1.2 and distal-Na_V_1.6. Surprisingly, in addition to the expression of Na_V_1.6, Na_V_1.2 was also found accumulated at a subpopulation of nodes of Ranvier in adult human cortex. Furthermore, we found that PV-positive cartridges, presumably formed by axon terminals of chandelier cells, mainly innervate the Na_V_1.6-enriched regions of the AIS.

## MATERIALS AND METHODS

### ETHICS STATEMENT

The procedures for handling and using the human brain tissue had been approved by the Biomedical Research Ethics Committee of Shanghai Institutes for Biological Sciences (license no. ER-SIBS-221004). The human brain tissues were obtained from the Shanghai Quyang Hospital. These tissues had to be removed surgically for the treatment of intractable epilepsy, cerebral hemorrhage, or brain tumor. Before the surgery all patients had signed a written consent form that described the experimental usage of their discarded tissues. The use and care of animals followed the guidelines of the Animal Advisory Committee at the Shanghai Institutes for Biological Sciences.

### TISSUE PREPARATION

#### Human cortical tissue

Brain tissues from 15 patients were used in this study, 10 with temporal lobe epilepsy, 1 with frontal lobe epilepsy, 3 with brain tumor, and 1 with cerebrovascular disease. The brain region and medical record for each patient were listed in **Table 1**. **Table 2** indicates the usage of tissue samples in different figures. Right after the tissue removal during surgery, the discarded human brain tissues were immediately immersed into an ice-cold oxygenated (95% O_2_ and 5% CO_2_) transportation solution containing (in mM) 213 sucrose, 2.5 KCl, 2 MgSO_4_, 2 CaCl_2_, 26 NaHCO_3_, 1.25 NaH_2_PO_4_, and 10 dextrose (315 mOsm, pH 7.4). The transportation of these tissues from the hospital to the laboratory usually took approximately 45 min. Cortical tissues were trimmed into small blocks, some of which were fixed directly and the rest stored in liquid nitrogen. We chose the surrounding tissues of surgically removed brain blocks that contained the tumor tissue or epileptic foci identified using electroencephalography (EEG) recordings. NeuN staining, Nissl staining, and/or AnkG staining were employed for tissue selection. Selected tissues should be histologically normal, showing no obvious decrease or increase (e.g., gliosis) in cell density, and exhibiting clear layer structure from pia to L6 and white matter. Tissues that did not meet the selection standards were not used in this study. Cortical blocks were kept in fixative for 2–4 h and dehydrated in 30% sucrose in 0.1 M PB for at least 24 h and then cut into sections with a thickness of 16–20 μm using a freezing microtome (Microm HM525, Thermo Scientific). The sections were collected and mounted on slides for the following staining procedures. In some experiments, we used brain blocks stored in liquid nitrogen. The blocks were transferred to the freezing microtome and cut into sections at a temperature of -14°C. The sections were then fixed with 0.5% paraformaldehyde (PFA) and 0.5% sucrose for 15 min. In general, this procedure would lead to a better staining of Na^+^ channels.

**Table 1:** Summary of clinical and surgical data.

No.	Age (yr), sex, side	Duration (yr)	Possible precipitating event	Seizure type	Seizure frequency (times/week)	Status epilepsy	Surgery removal area
1	5, F, R	2	Unknown	PC, gen	0.5–0.75	No	Anterior temporal lobe
2	25, M, R	1	Pilocytic astrocytoma	PC	1.5–14	No	Anterior temporal lobe
3	6, M, R	6	Cyst	PC, gen	1	No	Anterior temporal lobe
4	41, F, L	4	Unknown	PC, gen	2–2.5	No	Anterior temporal lobe
5	18, M, R	9	Birth problem	PC, gen	7–14	No	Anterior temporal lobe
6	42, M, L	18	High fever	PC, gen	7–35	No	Anterior temporal lobe
7	52, M, L	20	Cyst	PC	1–2	No	Anterior temporal lobe
8	24, M, R	11	Meningioma	PC, gen	1	Yes	Anterior temporal lobe
9	44, M, R	4	Hemangioma	PC, gen	3–14	No	Anterior temporal lobe
10	23, F, R	7	Unknown	PC, gen	0.5–1	No	Anterior portion of the medial and inferior frontal gyrus
11	22, F, R	20	Encephalitis	PC	14–35	Yes	Anterior temporal lobe

**Non-epilepsy control**			**Disease type and location**				**Surgery removal area**
12	57, F, R	10	Neurilemmoma in the middle cranial fossa				Anterior temporal lobe
13	79, M, L	2 h	17pcLacunar infarction of bilateral basal ganglia and right thalamus Brain hemorrhage of left thalamus				Junction area of temporal, parietal and occipital lobe
14	52, M, R	0.5	17pcGlioma in the junction area of parietal and occipital lobe and in the corpus callosum				Junction area of parietal and occipital lobe
15	45, M, L	5	Recurrent tumor in the frontal lobe				Frontal lobe

*M, male; F, female; R, right; L, left; PC, partial complex; PC, gen, partial complex, sometimes secondarily generalized.*

*Status epilepsy, epileptic attack lasting for more than 30 min (including the duration of consciousness loss if applicable).*

**Table 2:** Summary of tissue samples used for different figures.

Figure	Patient no. in Table 1	Sample size (*n*)	Figure	Patient no. in Table 1	Sample size (*n*)
**Figure 3**	Epi: 1,2,4,5,7	5	**Figure 5D**	Epi: 10,11	2
	Ctrl: 12–15	4	**Figure 6A**	Epi: 2,4,5,8,9,11	6
**Figures 4A–E**	Epi: 2,4,5	3		Ctrl: 12–15	4
	Ctrl: 12–15	4	**Figures 6B–D**	Epi: 2,4,5	3
**Figures 4F–G**	Epi: 8,9,11	3		Ctrl: 12–15	4
**Figure 5A**	Epi: 2,4,5,7	4	**Figure 7A**	Epi: 3,5,6,8	4
	Ctrl: 12–15	4	**Figure 7B**	Epi: 6,8	2
**Figures 5B–C**	Epi: 2,4,5	3	**Figures 7C–G**	Epi: 3,5,6	3
	Ctrl: 12–15	4			

*Epi, epileptic group; Ctrl, non-epileptic group.*

#### Rodent cortical tissue

Male SD rats (P30-P35) and Scn8a knock-out (KO) mice (Na_V_1.6^-/-^; P17; from the Jackson Lab) were perfused as described previously (Hu et al., 2009). Briefly, animals were anesthetized with sodium pentobarbital (40 mg/kg, i.p.) and showed no avoidance response to foot pinch. They were then perfused with normal saline (at 37°C) and followed by ice-cold fixative. The brain was post-fixed, dehydrated in 30% sucrose solution, and cut into sections. In some experiments, we also performed decapitation after anesthesia (without perfusion), dissected out the brain, and then cut into sections using the freezing microtome. Although the staining patterns of Na^+^ channels with the two procedures were similar, more robust signals were found in sections without animal perfusion.

#### Fixative

Different fixatives were used for various staining experiments. Protocols including fixation methods and the fixative compositions were described in **Table 3**.

**Table 3:** Different fixation protocols for immunostaining.

Double or triple staining	Fixative	Fixation and cutting procedures
**Human**
Pan-Na_V_(R),NeuN(M), AnkG(G) Na_V_1.2(R), NeuN (M), AnkG(G) Na_V_1.6(R), NeuN (M), AnkG(G) Na_V_1.1(R), AnkG(G)	0.5%	Freezing sectioning of unfixed tissue blocksFixation for 15 min.
PV (R), NeuN (M), AnkG(G) Na_V_1.6(R), MBP (M), AnkG(G)	4%	Block fixation for 2–4 h and sucrose dehydration
GAT-1 (R), PV (M), V-GAT (GP) Na_V_1.6(R), PV (M), AnkG(G) Na_V_1.6(R), AnkG(G)	2%	Freezing sectioning.
**Mice**
NaV1.6(R), AnkG(G)	0.5%	Animal perfusion Post fixation for 1.5–3 h and sucrose dehydration Freezing sectioning.
**SD rats**
Na_V_1.2(R),Na_V_1.2 (M), AnkG(G)	0.5%	Freezing sectioning of unfixed tissues; Fixation for 15 min.

*AnkG, Ankyrin G; M, mouse; Rb, rabbit; G, goat; GP, Guinea pig.*

*Note that all fixatives contain equal concentrations of paraformaldehyde (PFA) and sucrose.*

### FLUORESCENCE IMMUNOHISTOCHEMISTRY

We rinsed the sections in 0.01 M phosphate-buffered saline (PBS, pH 7.4) and incubated them first with 0.3% Triton X-100 in PBS for 30 min and then with the blocking solution (10% BSA) for 1 h. Sections were incubated overnight at room temperature with the primary antibodies diluted in 0.1% TritonX-100 and 10% BSA. The following primary antibodies were used: mouse anti-Na_V_1.1 (73-023; 1:200; NeuroMab), rabbit anti-Na_V_1.2 (Rb-Na_V_1.2; ASC-002; 1:400; Alomone Labs), mouse anti-Na_V_1.2 (M-Na_V_1.2; 73-024; 1:200; NeuroMab), rabbit anti-Na_V_1.6 (ASC-009; 1:400; Alomone Labs), rabbit anti-Pan-Na_V_ (ASC-003; 1:400; Alomone Labs), mouse anti-PV (MAB1572; 1:1000; Millipore), rabbit anti-PV (PV-25; 1:1000; Swant), mouse anti-MBP (SMI-94R; 1:400; Covance), mouse anti-NeuN (MAB377; 1:1000; Millipore), goat anti-Ankyrin G (AnkG; sc-31778; 1:400; Santa cruz), mouse anti-AnkG (sc-12719; 1:400; Santa cruz), guinea pig anti-vesicular GABA transporter (V-GAT; 131004; 1:100; Synaptic Systems), rabbit anti-GABA transporter-1 (GAT-1; AB1570; 1:400; Millipore), mouse anti-contactin-associated membrane protein (anti-Caspr; 75-001; 1:500; NeuroMab). After complete wash, sections were incubated for 2 h in the secondary antibodies (1:1000, Invitrogen): Alexa 488 conjugated donkey anti-rabbit, Alexa 555 conjugated donkey anti-mouse, and Alexa 647 conjugated donkey anti-goat, or Alexa 488 conjugated donkey anti-guinea pig, Alexa 555 conjugated donkey anti-mouse, and Alexa 647 conjugated donkey anti-rabbit. Finally, the sections were washed and mounted with fluoromount-G (Electron microscopy science).

We took images using a laser scanning confocal microscope (ECLIPSEFN1, Nikon) with a 40× (NA 1.3) or 60× (NA 1.4) oil-immersion objective. The acquisition parameters were carefully adjusted to make the fluorescence signals linearly displayed and fall into the maximum dynamic range of the detector. Z-stack images were collected with a voxel interval of 0.5 or 1 μm. We then used Autoquant X2 software (Media Cybernetics) to deconvolve these images. We employed Fiji software (http://pacific.mpi-cbg.de) to obtain the maximum Z-stack projection and make appropriate adjustment of brightness and contrast.

### SPECIFICITY OF THE ANTIBODIES

We examined the specificity of antibodies to make sure the stainings were true positive. Omission of primary antibodies from the immunostaining protocols resulted in undetectable signals for all antibodies examined. We further tested the specificity of Na_V_1.6 antibody with transgenic mice. Na_V_1.6 immunosignals were absent from the AIS in the Na_V_1.6^-/-^ mice, whereas robust staining could be observed in the control littermates (Figures S1A,B in Supplementary Material, P17, *n* = 2 pairs of mice). In control mice (Na_V_1.6^+/+^), Na_V_1.2 immunosignals could be observed at proximal regions of the AIS and showed spatial segregation with Na_V_1.6 signals (Figure S1A in Supplementary Material), a distribution pattern similar to previous findings in rats (Hu et al., 2009). In the Na_V_1.6^-/-^ mice, distribution of Na_V_1.2 immunosignals became relatively even along the AIS, possibly a compensatory response to Na_V_1.6 loss (Figure S1B in Supplementary Material). We did observe some thin fibers positive for Na_V_1.6 in the Na_V_1.6^-/-^ mice; however, they were rarely observed and showed no co-labeling with AnkG. Despite these rare false positive staining, the lack of robust immunosignals in Na_V_1.6^-/-^ mice and the presence of strong signals colocalized with AnkG in wild type mice indicate a high specificity of the Na_V_1.6 antibody.

For Na_V_1.2 antibody, it was not possible to test the antibody specificity in transgenic mice because the Na_V_1.2 KO mice were lethal perinatally (Planells-Cases et al., 2000). Alternatively, we co-labeled two antibodies against different peptide sequences of rat Na_V_1.2 to examine their specificity, one polyclonal antibody against the intracellular loop between domain I and II (467–485) generated from rabbit (Rb-Na_V_1.2), and the other monoclonal antibody against cytoplasmic C-terminal (1882–2005) from mouse (M-Na_V_1.2). Immunosignals stained with the two antibodies overlapped well with each other in rat neocortical sections (Figure S1C in Supplementary Material, *n* = 2 rats), indicating a high specificity of the two antibodies. For the Rb-Na_V_1.2 antibody, preabsorption with its antigen for 2 h could completely block the staining, indicating that the staining with this antibody was specific (Figure S1D in Supplementary Material, *n* = 2 rats). Usually, M-Na_V_1.2 immunosignals were more robust than those of Rb-Na_V_1.2 in rodent tissue. In the human neocortex, however, only Rb-Na_V_1.2 antibody showed strong stainings, whereas M-Na_V_1.2 antibody exhibited no detectable signal in our experiments. This failure of M-Na_V_1.2 antibody in staining the human tissue could be attributed to differences in antigen peptide sequences (1882–2005) of Na_V_1.2 protein between rat and human. Therefore, we chose to use Rb-Na_V_1.2 for human tissue experiments.

Although we did not examine the specificity of Na_V_1.1 antibody as strictly as that of Na_V_1.2 and Na_V_1.6, its staining pattern in rodents was in agreement with previous findings (Yu et al., 2006; Ogiwara et al., 2007; Lorincz and Nusser, 2008). In our experiments, Na_V_1.1 immunosignals could be detected only in GABAergic interneurons in the rat neocortex; in addition, blocking peptide could completely block the staining, suggesting a high antibody specificity (data not shown). The accumulation of Na^+^ channels at the AIS and nodes of Ranvier is achieved through anchoring to the actin cytoskeleton via AnkG and other proteins, and AnkG is therefore usually used as a marker of the AIS and the nodes along the axon (Srinivasan et al., 1988; Kordeli et al., 1995; Zhou et al., 1998). The Caspr is another molecule that can be used to define the paranodal regions at the nodes (Einheber et al., 1997; Menegoz et al., 1997; Peles et al., 1997). In this study, Caspr together with AnkG were used as markers to identify the nodes of Ranvier. The immunosignals of two AnkG antibodies against different antigen epitopes showed complete overlap at the AIS and at the nodes of Ranvier (Figure S2 in Supplementary Material). We normally used AnkG antibody generated from goat unless otherwise specified.

### QUANTITATIVE ANALYSIS

We analyzed the immunostaining signals in neurons whose AIS emitted directly from the soma (labeled by NeuN antibody) and extended for more than 40 μm in length. For each patient, we acquired images (no less than six images, 215 μm × 215 μm) from at least three cortical sections. For quantitative analysis of the fluorescence intensity along the AIS, we extracted the fluorescence signals at the AIS (for Na^+^ channels) or those surrounding the AIS (for PV signals) with the Fiji software, and calculated the fluorescence intensity in three-dimension with the Amira software (Visualization Sciences Group). Firstly, we used Autoquant X2 software to deconvolve images, and Fiji software to combine the two images of Na^+^ channel and AnkG into one 8-bit image. We then traced AIS in this image, filled out the AIS paths with different radius for Na^+^ channels and PV signals, and obtained the mask image of this fillout. We performed an AND operation of the mask image and the individual image of Na^+^ channel or PV to obtain an image with fluorescence signals only at or surrounding the AIS but without any other background noise. Next, we calculated the fluorescence intensity along the AIS with Amira. For individual AIS, we averaged the fluorescence intensity every 1 μm and normalized these values to the maximum average intensity with MATLAB (MathWorks). Finally, we obtained the distribution pattern of fluorescence intensity along the AIS by averaging the normalized values of different AIS.

To examine the percentage of nodes expressing a certain Na^+^ channel subtype in the white matter of the cortex, we performed analysis on images double stained with AnkG and channel antibodies. We counted the number of nodes positive for Na_V_1.2, Na_V_1.6, or Pan-Na_V_ in a square area (160 μm × 160 μm) and normalized the number to the total pixel number of AnkG signals with fluorescence intensity higher than a threshold value (mean + SD). For each double-staining group, six such square areas from three human patients (two from each patient) were analyzed and the normalized values were then averaged. Because Pan-Na_V_ antibody recognizes all Na^+^ channel subtypes, it should label all the nodes of Ranvier in the cortex. We divided the average value for Na_V_1.2 or Na_V_1.6 by that of Pan-Na_V_ to obtain the percentage of nodes containing Na_V_1.2 or Na_V_1.6 in the cortex.

For a particular experiment, we carried out immunostaining in different batches, but with the same protocol. For quantitative analysis of the immunosignals of PV and Na^+^ channels, we compared the normalized fluorescence intensity and examined their distribution pattern along the AIS. The absolute Na^+^ channel density could not be determined in our study.

## RESULTS

### MOLECULAR IDENTITY OF AIS Na^+^ CHANNELS IN HUMAN PCs

**FIGURE 1:**
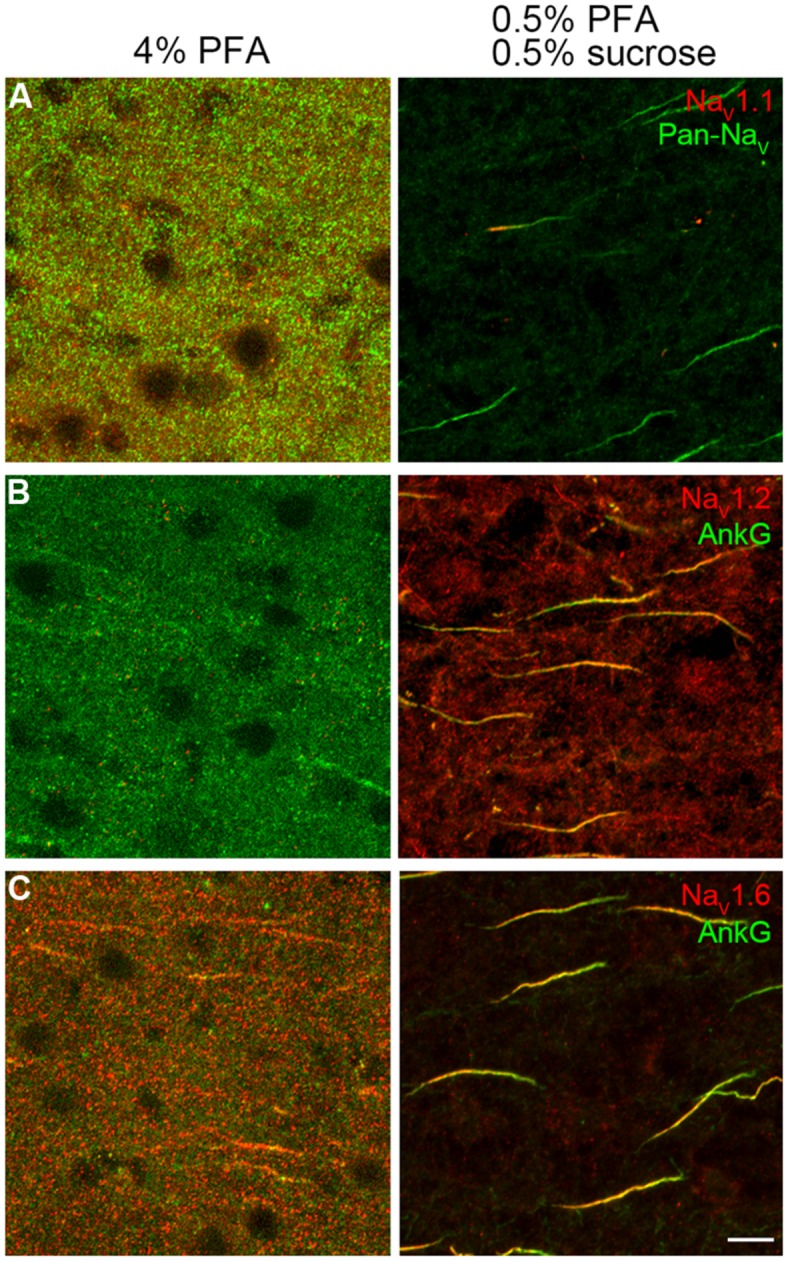
**Paraformaldehyde (PFA) concentration was crucial for Na^+^ channel immunostaining. (A–C**, left column) Conventional fixative (4% PFA) revealed no specific labeling except a few immunosignals of Na_V_1.6 and AnkG with extremely low signal-to-noise ratio in the prefrontal cortex of rats. (**A–C**, right column), light fixation (0.5% PFA and 0.5% sucrose) resulted in robust stainings of all antibodies examined (Na_V_1.1, Pan-Na_V_, Na_V_1.2, Na_V_1.6, and AnkG, color-coded). The voxel depth is 4 μm in **(A)**, 3 μm in **(B)**, 5 μm in **(C)**. Scale bar, 10 μm. Pia locates to the right of the images.

For Na^+^ channel staining, conventional immunohistochemical reactions using 4% PFA revealed almost no labeling (**Figures 1A–C**, left panels), while addition of sucrose (4%) would lead to a weak staining of Na_V_1.6 channels in the AIS (data not shown). Reducing the concentrations of both PFA and sucrose to 0.5% revealed a better staining. This weak fixative (0.5% PFA and 0.5% sucrose) ensured a satisfactory staining with a high signal-to-noise ratio of all antibodies against Na^+^ channels used in this study (**Figures 1A–C**, right panels). This result suggests that the conventional fixative is relatively strong and possibly masks the antigen epitope.

**FIGURE 2:**
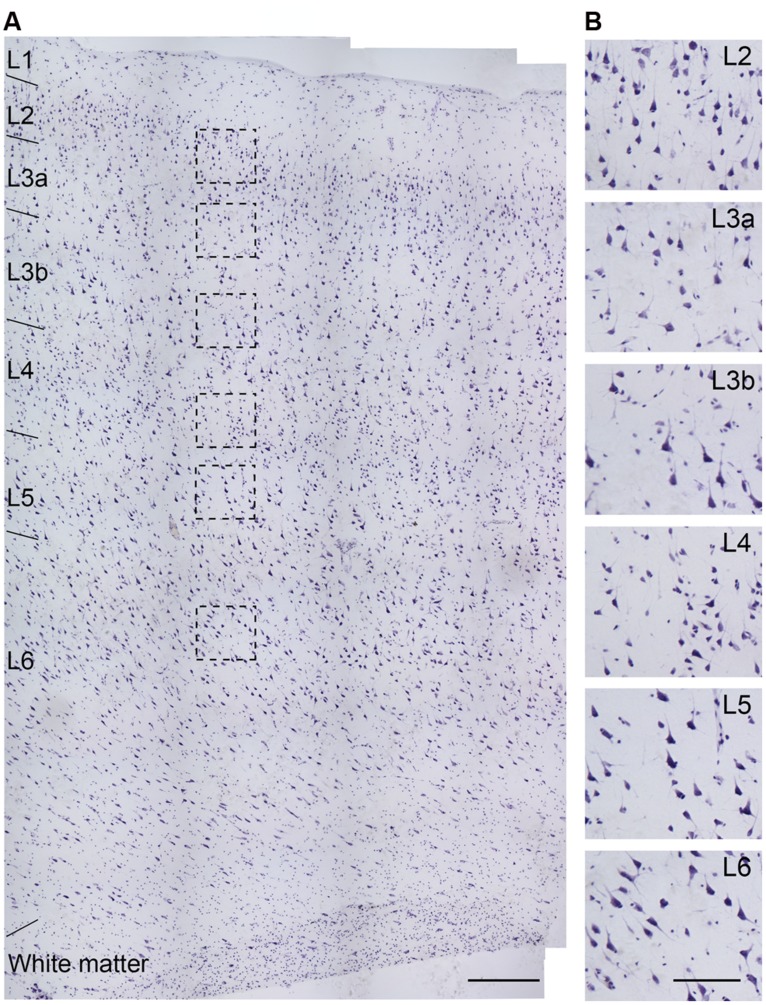
**Nissl staining of a section obtained from the temporal lobe of an epileptic patient (No. 5 in Table 1). (A)** Cortical layers could be easily distinguished by cell size, shape, and density. **(B)** High-power photomicrograph of the boxed areas in **(A)**. The cell density in L3a is lower than that in L2, but slightly higher than that in L3b, while the somatic size of L3b PCs is bigger than that in L3a. Note the higher density of cells with smaller soma size in L4 than other layers. Scale bars represent 300 μm in **(A)**, and 100 μm in **(B)**.

To investigate the molecular identity of Na^+^ channels distributed at the AIS of human cortical PCs, we performed immunostaining in cortical tissues obtained from patients with intractable temporal lobe epilepsy (*n* = 5), cerebrovascular disease (*n* = 1), and brain tumor (*n* = 3). Usage of the tissue samples were listed in **Table 2**. We used peri-foci or peri-tumor cortical tissues that showed clear layer structure (from L1 to L6 and the white matter) and the densities of NeuN-, Nissl-, or AnkG-positive signals were not obviously reduced (**Figure 2**). In our experiments, we employed Pan-Na_V_ antibody that could recognize all α subunits to reveal the total density of Na^+^ channels. NeuN and/or AnkG immunosignals were used to distinguish PCs from interneurons. Neurons with NeuN-positive and pyramidal-shaped somata and thick AnkG-positive AIS were considered as putative PCs. In general, the AIS of PCs uniformly projected toward deep layers. In comparison with PCs, the somata of interneurons were usually smaller and multi-shaped, and their AIS were thinner and showed unpredictable projection directions (Figure S3 in Supplementary Material).

**FIGURE 3:**
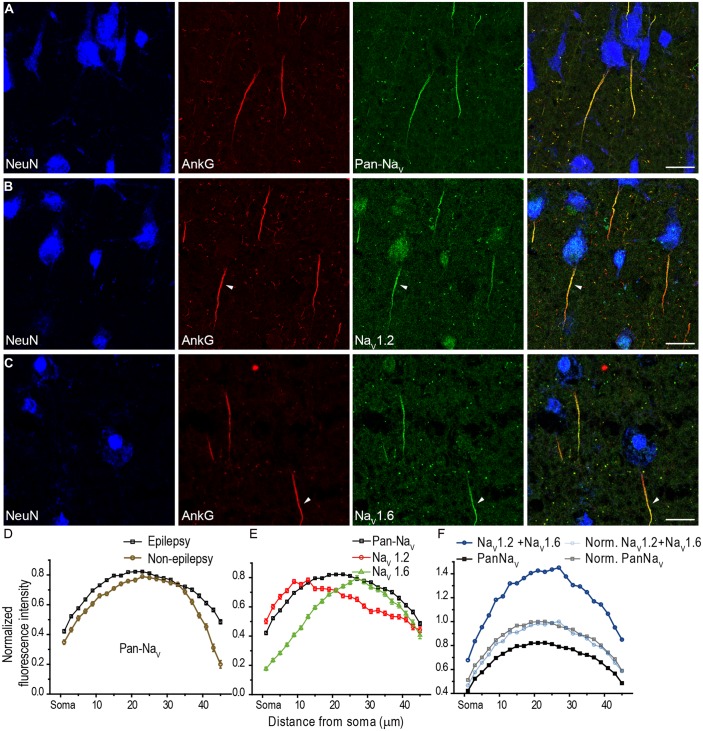
**Distribution of Na^+^ channel subtypes at the AIS of human cortical L3 PCs. (A)** Representative confocal images showing triple staining of NeuN (blue), AnkG (red), and Pan-Na_V_ (green). Note that Pan-Na_V_ immunosignals were intense along the entire AIS. **(B,C)** Triple staining of NeuN, AnkG, and Na_V_1.2 (or Na_V_1.6). Note that Na_V_1.2 immunosignals **(B)** occupied the proximal portion of the AIS (arrowhead), while Na_V_1.6 signals **(C)** mainly located at the distal region of AIS (arrowhead). **(D–F)** Plots of the average normalized fluorescence intensity (mean ± SEM, see Section Material and Methods) as a function of distance at the AIS from the soma. The depth of Z-projected image is 2.5 μm in **(A)** and **(B)**, 3 μm in **(C)**. Scale bars represent 20 μm.

In agreement with previous studies (Inda et al., 2006), our experiments showed a high density of Na^+^ channels at the AIS of human PCs (**Figure 3**). Strong fluorescence signals of Pan-Na_V_ were detected along the entire AIS (**Figure 3A**). We plotted the fluorescence distribution of Pan-Na_V_ in PCs of L3 (L3a and L3b) and found that the intensity remained high from the proximal to the distal AIS except for the two ends (the proximal end, 0–7 μm from the soma; and the distal end, 41–45 μm) where the intensity was relatively low (50% < intensity < 80% of the peak intensity, *n* = 113 from five epileptic subjects; **Figures 3A,D**). The distribution profile of the normalized Pan-Na_V_ intensity in epileptic tissues was similar to that in non-epileptic tissues (*n* = 62 from four non-epileptic subjects; **Figure 3D**), suggesting no substantial influence of epileptic seizures on the distribution pattern of total Na^+^ channels. In the following experiments, we mainly used cortical tissues from epileptic patients. No fluorescence signals could be found at the soma and proximal dendrites (stained by NeuN), suggesting an extremely low density of Na^+^ channels at these compartments. In these experiments, although the quality of the NeuN labeling was variable in tissues fixed after freezing sectioning (**Figures 3A–C**), the shape of the soma could still be easily distinguished.

At the AIS of cortical PCs, both Na_V_1.2 and Na_V_1.6 immunosignals were observed, but they accumulated at different compartments. Similar spatial segregation of channel subtypes could be found in PCs of pediatric epileptic patients, we therefore pooled the data together with adult patients. In L3b, Na_V_1.2 signals concentrated at the proximal AIS region with fluorescence intensity peaked at 9–14 μm from the soma (*n* = 57 from five epileptic subjects, **Figures 3B,E**), whereas Na_V_1.6 immunosignals accumulated at the distal AIS with the peak intensity at 25–29 μm from the soma (*n* = 62, **Figures 3C,E**). Although there was an overlap between Na_V_1.2 and Na_V_1.6 immunopositive regions, a clear segregation of these channel subtypes was found at the AIS. Interestingly, only when the distribution curves of Na_V_1.2 and Na_V_1.6 were respectively multiplied by a same factor, their summation curve could show a similar distribution profile with that of Pan-Na_V_ curve (**Figure 3F**), which could be reflected by a perfect overlap of the two curves after further normalization (**Figure 3F**). Together, these results indicate a spatial segregated distribution of proximal-Na_V_1.2 and distal-Na_V_1.6 at the AIS of human cortical PCs, similar to the segregated distribution of the two channel subtypes in rat PCs (Hu et al., 2009).

**FIGURE 4:**
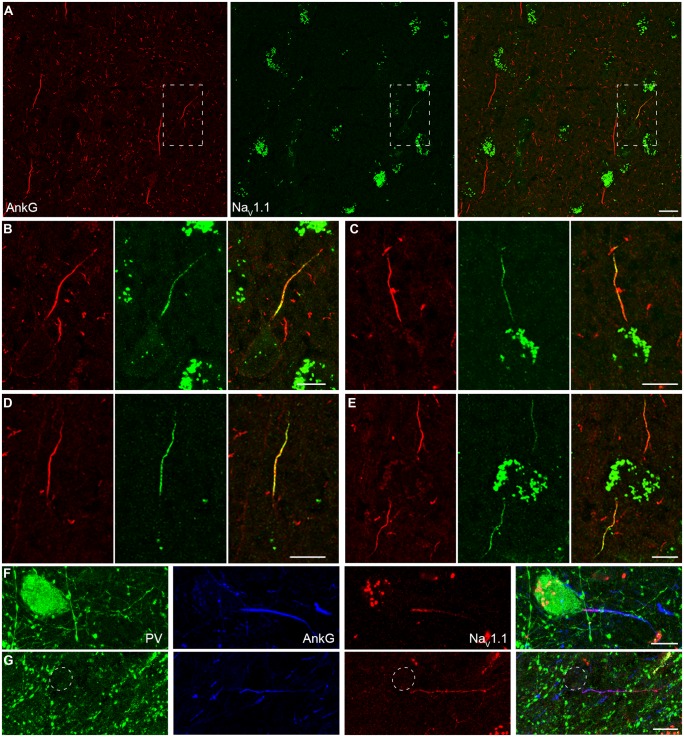
**Distribution of Na_V_1.1 at the AIS of interneurons in L2–L6. (A)** Double staining of AnkG (red) and Na_V_1.1 (green) showed that positive stainings of Na_V_1.1 were only found in the thin AIS of a putative interneuron (boxed area). Note that the orientation and thickness of the AIS were different from those of putative PCs. **(B)** High magnification of the boxed area shown in A. Na_V_1.1 immunosignals were more concentrated at the proximal AIS. **(C–E)** Other examples of Na_V_1.1 stainings at the AIS of interneurons. The distribution pattern and the relative fluorescence intensity varied across interneurons. Note that the two AIS in E showed different fluorescence intensity. **(F–G)** Triple staining of PV (green), AnkG (blue), and Na_V_1.1 (red). **(F)** Na_V_1.1 immunosignals accumulated at the AIS of the PV-positive neuron, especially at the very proximal AIS region. **(G)** In a putative interneuron (PV-negative), Na_V_1.1 signals could be also found at the AIS, but distributed evenly. Note the axon trunk beyond the AIS was also stained. Pia locates to the top in **(A–E)**, and to the left in **(F**,**G)**. Thickness of the Z-stacks is 3.5 μm in **(A)**, 3 μm in **(B,D,E)**, 1 μm in **(C)**, 5 μm in **(F)**, 8 μm in **(G)**. Scale bars represent 20 μm in **(A)**, 10 μm in **(B**–**G)**.

In addition to Na_V_1.2 and Na_V_1.6, Na_V_1.1 is another main Na^+^ channel subtype in adult CNS. Na_V_1.1 was revealed to be expressed in the axon of cortical interneurons in rodents (Ogiwara et al., 2007; Lorincz and Nusser, 2008); however, in human cortex, Na_V_1.1 was reported to be distributed in PCs (Whitaker et al., 2001b). In all human tissues we examined, fluorescence signals of Na_V_1.1 were only observed at the AIS of putative interneurons whose AnkG-labeled AIS were usually thin and projected unpredictably; in contrast, they were completely absent from thick AIS of putative PCs (**Figure 4A**). AIS with Na_V_1.1 immunosignals could be found in layers from L2 to L6. Moreover, the staining intensity and distribution pattern of Na_V_1.1 diversified across different interneurons (**Figures 4B–E**). Na_V_1.1 channels could accumulate at either the proximal or the distal portion of the AIS (**Figures 4B,C**), and sometimes express uniformly along the AIS (**Figure 4D**). The intensity of Na_V_1.1 immunosignals were obviously different in the two neighboring interneurons shown in **Figure 4E**. With triple staining of PV, Na_V_1.1, and AnkG, we found that PV-positive neurons showed Na_V_1.1 immunosignals at their AIS, especially concentrating at a short compartment very close to the soma (**Figure 4F**). In PV-negative interneurons, we also found Na_V_1.1 signals at their AIS (**Figure 4G**), but they showed different distribution patterns. Therefore, distinct Na_V_1.1 distribution profiles might be attributed to differences in interneuron cell types. However, we could not exclude the possibility that the distribution patterns of Na_V_1.1 may be subject to change in response to epileptic seizures. These results indicate that Na_V_1.1 channels are expressed by human inhibitory interneurons, but its distribution properties in different kind of interneurons remain to be further examined.

Together, our results reveal a high density of Na^+^ channels and a segregated distribution of channel subtypes at the AIS, with Na_V_1.2 and Na_V_1.6 channels accumulating at the proximal and the distal AIS of human PCs, respectively. In addition, unlike those of putative interneurons, the AIS of PCs had no detectable immunosignals for Na_V_1.1. Due to the critical roles of Na^+^ channels in regulating AP generation and neuronal excitability, the presence of Na_V_1.1 at the AIS of PV-positive neurons suggests a role of this channel subtype in regulating the excitability of inhibitory interneurons and thus the excitation–inhibition balance in cortical networks. Indeed, previous studies found severe spontaneous epileptic seizures in loss-of-function mutations of Na_V_1.1 gene in human (Baulac et al., 1999; Escayg et al., 2000; Claes et al., 2001; Meisler and Kearney, 2005; Catterall et al., 2010).

### CHANNEL SUBTYPES DISTRIBUTED AT NODES OF RANVIER

**FIGURE 5:**
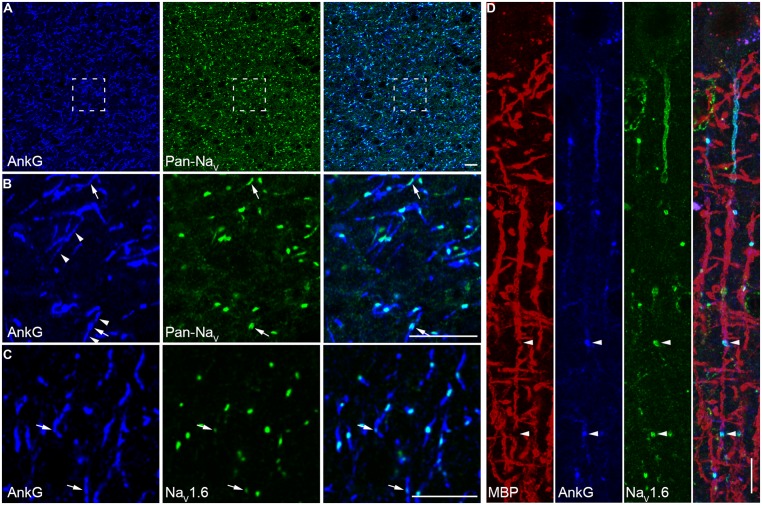
**A high density of Na_V_1.6 distributes at the nodes of Ranvier. (A)** Double staining of AnkG (blue) and Pan-Na_V_ (green) in the white matter of human neocortex. **(B)** High magnification of the boxed area shown in A. Robust Pan-Na_V_ immunosignals were found restricted to small regions of AnkG-labeled nodes of Ranvier. The AnkG immunosignals at the nodes showed different intensity (arrow). Detectable AnkG signals were also located at the two flanking regions of the nodes (arrowheads). **(C)** Co-labeling of AnkG and Na_V_1.6 (green) showing that distribution patterns of Na_V_1.6 were similar to those of Pan-Na_V_ as shown in **(B)**, but note that at some nodes the Na_V_1.6 signals were very weak (arrows). **(D)** Triple staining of MBP (red), AnkG (blue), and Na_V_1.6 (green) in L2/3. The Na_V_1.6 signals occupied the nodes of Ranvier (arrowheads) interspersed between the MBP-positive structures in cortical layers. The thickness of Z-stacks is 4 μm in **(A,B)**, 3 μm in **(C)**, 2.5 μm in **(D)**. Scale bars represent 10 μm.

At the myelinated axon beyond the AIS, AP is generated at the nodes of Ranvier and propagates down the axon in a saltatory mode. This saltatory conduction ensures rapid AP conduction and fast communication between neurons. In rodents, a high density of Na_V_1.6 was found at the nodes of Ranvier (Caldwell et al., 2000). Here we examined the molecular identity of nodal Na^+^ channels in the human neocortex. Double staining in the white matter revealed a high density of nodes labeled with strong immunosignals of AnkG and Pan-Na_V_ (**Figures 5A,B**). Detectable AnkG signals were also observed at the paranodal regions where AnkG signals colocalized with Caspr (Figure S4A in Supplementary Material). Some nodes displayed very low AnkG signals, and the fluorescence intensity could be even lower than that in the flanking regions (**Figure 5B**). Moreover, we also observed gaps between labeled nodal and paranodal regions (Figure S2B in Supplementary Material). These observations may suggest a heterogeneous population of nodes of Ranvier. To disclose the molecular identity of Na^+^ channel subtypes at the nodes, we performed double staining using AnkG and Na_V_1.6 antibodies. Strong Na_V_1.6 signals were observed at the nodes (**Figure 5C**), similar to those found in rodents. The Na_V_1.6-positive puncta could be also found in the gray matter (**Figure 3C**), especially in deep layers. To ensure that these puncta were indeed the nodes of Ranvier, we used MBP antibody to visualize the myelination of axons. Triple staining of Na_V_1.6, AnkG, and MBP showed that puncta positive for both Na_V_1.6 and AnkG interspersed between MBP-labeled myelin sheaths and confirmed their nodal identity (Figures S4B,C in Supplementary Material and **Figure 5D**).

**FIGURE 6:**
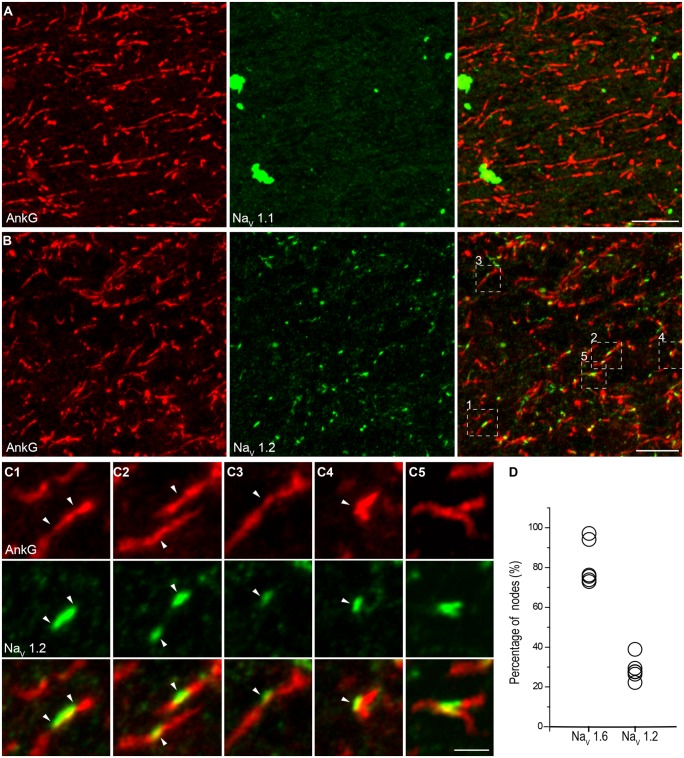
**Na_V_1.2 but not Na_V_1.1 channels accumulate at the nodes of Ranvier in adult human cortical tissue. (A)** Double staining of AnkG (red) and Na_V_1.1 (green) in the white matter indicating the absence of Na_V_1.1 at the nodes. **(B)** Double labeling of AnkG and Na_V_1.2 (green). Note that Na_V_1.2 positive puncta scattered in the white matter and colocalized with segmental AnkG-positive axon processes, indicating these puncta were nodes immunoreactive to Na_V_1.2. There were also AnkG-positive nodes without Na_V_1.2 expressed. **(C)** High-magnification images of the box areas shown in **(B)**. These individual nodes **(C1–C5)** expressed Na_V_1.2 but showed diversification in their length, shape and labeling pattern. **(D)** Group data of the percentage of nodes with different Na^+^ channel subtypes. The thickness of Z-stacks is 2 μm in **(A)**, 6 μm in **(B,C2)**, 3 μm in **(C1**,**C3,C4)**, 1 μm in **(C5)**. Scale bars represent 10 μm in **(A,B)**, and 2 μm in **(C)**.

In double staining of Na_V_1.6 and AnkG, some nodes showed very weak Na_V_1.6 immunosignals, or even no detectable signals (**Figure 5C**). Considering that this kind of nodes was rarely observed in the Pan-Na_V_ staining, we speculated that other channel subtypes (Na_V_1.1 and Na_V_1.2) may also express at the nodes. However, no Na_V_1.1 signal was found at any of the AnkG-positive nodes in both the white matter (**Figure 6A**) and the gray matter (**Figure 4A**). Previous studies in the CNS of rodents showed that Na_V_1.2 channels expressed at early developmental stages but were replaced by Na_V_1.6 as the axon became myelinated (Boiko et al., 2001; Kaplan et al., 2001). Surprisingly, in adult human cortex, Na_V_1.2 immunosignals were observed at AnkG-positive nodes in both the white matter (**Figure 6B**) and the gray matter (**Figure 3B**) of epileptic and non-epileptic tissues. The Na_V_1.2-positive nodes showed diversity in terms of their length, shape and labeling patterns (**Figures 6C1–C4**). As shown in **Figure 6C5**, Na_V_1.2 immunosignals were occasionally found at or around branching points of AnkG-labeled processes. The percentages of Na_V_1.6- and Na_V_1.2-positive nodes in the white matter were 81.6 ± 4.5% and 28.7 ± 2.3% respectively (Na_V_1.6: *n* = 8127 nodes; Na_V_1.2: *n* = 2978 nodes; Pan-Na_V_: *n* = 10768 nodes from three human subjects). Because both Na_V_1.2 and Na_V_1.6 antibodies were generated from rabbit, we were not able to perform double staining of these channels to examine their co-localization at individual nodes in the human tissue. The summated percentage of nodes expressing the two subtypes was more than 100%, suggesting a co-expression of Na_V_1.2 and Na_V_1.6 at some individual nodes; however, this requires further confirmation using appropriate antibodies.

Ion channel expression could be subject to change with the development of brain disorders, for example, demyelinated axon in multiple sclerosis could regain the expression of Na_V_1.2 (Craner et al., 2003, 2004). In our experiments, most of the human tissues were obtained from epileptic subjects; we therefore tried to examine the Na^+^ channel distribution in non-epileptic tissues. In addition to the similar distribution pattern of Pan-Na_V_ along the AIS between epileptic and non-epileptic groups (**Figure 3D**), the segregated distribution of proximal-Na_V_1.2 and distal-Na_V_1.6 was also observed in non-epileptic tissues. Similarly, we also found the presence of the two channel subtypes at the nodes of Ranvier and the absence of Na_V_1.1 at the axon (data not shown). Because the tissues from different patients were not processed simultaneously, we were not able to determine whether the fluorescence intensity of Na^+^ channel staining was subject to change in response to epilepsy. However, the current experiments suggest that the distribution pattern of channel subtypes could not be influenced by epileptic seizures.

### PV-POSITIVE CARTRIDGES SELECTIVELY INNERVATE THE Na_V_1.6-ENRICHED AIS DOMAIN

The AIS of PCs is innervated by axon terminals of GABAergic chandelier cells. This strategic innervation has been believed to play important roles in regulating AP generation at the AIS and further modulating the main neuronal output. Considering that Na_V_1.6 is the channel subtype that determines AP initiation, we next sought to investigate whether axon terminals of chandelier cells selectively targeted to the Na_V_1.6-enriched AIS segment.

**FIGURE 7:**
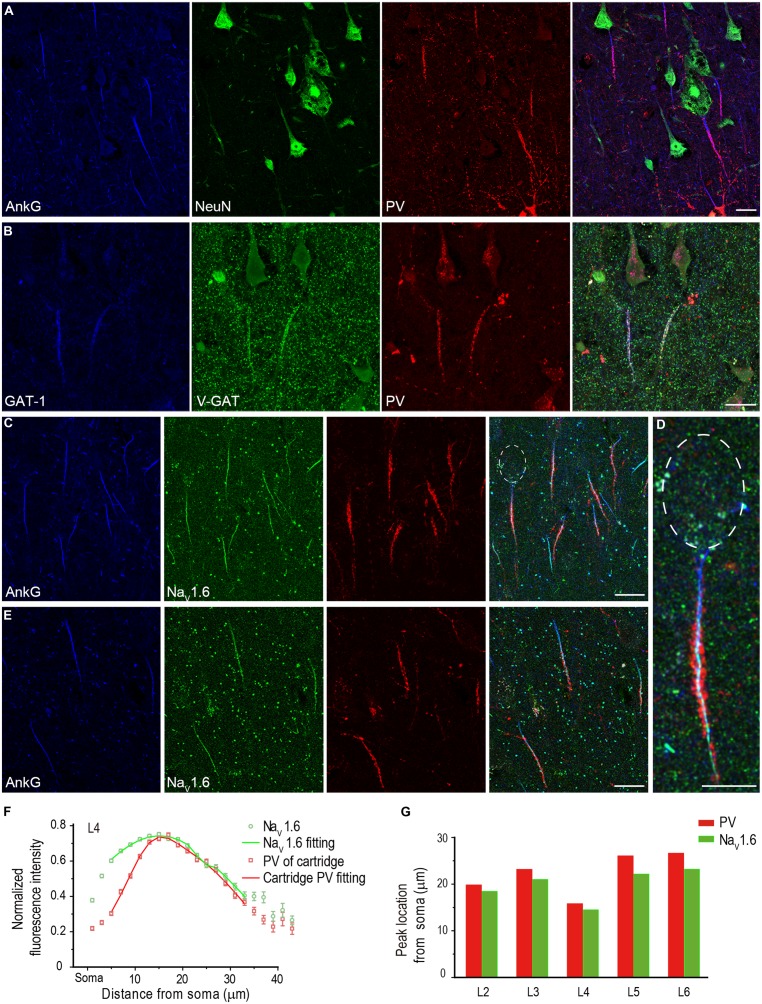
**PV-positive axon cartridges innervate Na_V_1.6-enriched distal AIS. (A)** Triple staining of AnkG (blue), NeuN (green), and PV (red) showing that PV-positive cartridge structure surrounded the AIS. **(B)** Triple staining of GAT-1 (blue), V-GAT (green), and PV. The colocalization of these molecules at the cartridge structure indicates that PV-positive cartridges were indeed inhibitory axonal arbors. **(C–E)** Triple staining of AnkG (blue), Na_V_1.6 (green), and PV in L4 **(C,D)** and L5 **(E)** showed that the PV-positive cartridges mainly targeted the Na_V_1.6-enriched AIS region. **(D)** Higher magnification of the individual neuron with the soma highlighted in **(C)**. Individual cartridges in L4 possessed more puncta than those in L5. **(F)** Plotting and fitting the normalized PV and Na_V_1.6 immunofluorescence intensity along the AIS of PCs in L4. Both PV and Na_V_1.6 reached their peak fluorescence intensity at 14–16 μm away from the soma. **(G)** Plotting of the peak-intensity location for PV and Na_V_1.6 across different cortical layers. The thickness of Z-stacks is 4 μm in A, 5 μm in **(B–E)**. Scale bars represent 20 μm in **(A–C,E)**, and 10 μm in **(D)**.

The specialized axonal arbors of chandelier cells, usually called cartridge, are formed by arrays of vertically oriented presynaptic terminals. In primates, chandelier cells were immunoreactive to PV, and immunostaining of PV was usually employed to visualize the cartridge structure (DeFelipe et al., 1989; Lewis and Lund, 1990), though a recent study suggested that not all chandelier cells expressed PV in rodents (Taniguchi et al., 2013). Indeed, vertically distributed PV-positive cartridges could be observed and they closely surrounded the AIS of putative PCs in the human neocortex (**Figure 7A**). Triple staining of GAT-1, PV, and VGAT indicated co-localization of these three molecules at the cartridge structure, suggesting the PV-containing cartridges are indeed GABAergic presynaptic terminals (**Figure 7B**). In comparison with VGAT, much less GAT-1-positive puncta were observed in structures outside cartridges, indicating that GAT-1 specifically locates at cartridges in the human neocortex. This result is also consistent with previous findings (DeFelipe and Gonzalez-Albo, 1998; Inda et al., 2006).

Because there was a fluorescence gap between PV-positive cartridge and the soma, we speculated that the cartridges selectively innervated the Na_V_1.6-enriched AIS domain. We performed triple staining of AnkG, PV, and Na_V_1.6, and measured the fluorescence intensity of cartridge PV and Na_V_1.6 along the entire AIS of PCs (*n* = 367 from three epileptic subjects) across cortical layers. Interestingly, PV-labeled cartridges in L4 showed few innervations to the proximal AIS; however, they covered the distal AIS region where Na_V_1.6 immunosignals were much stronger (**Figures 7C,D**). PV cartridges in L5 also innervated the Na_V_1.6-enriched distal AIS but with much less PV positive puncta than those in L4 (**Figure 7E**). Many Na_V_1.6-positive nodes could be easily found in these two layers. In L4, the location of the peak fluorescence intensity for cartridge PV (16 μm away from the soma, fitting with double Gaussian function, *n* = 142 from three epileptic subjects) was similar to that of Na_V_1.6 (15 μm; **Figure 7F**). At the proximal AIS, the normalized PV fluorescence intensity was relatively low but increased steeply until reaching its peak at distal AIS regions, indicating a more confined distribution of cartridge PV than that of Na_V_1.6. Distribution pattern of cartridge PV and Na_V_1.6 at and beyond the peak location were similar. Although the fluorescence intensity of Na_V_1.6 peaked differently at the AIS across cortical layers, their peak location matched well with that of PV cartridges (**Figure 7G**; L2: *n* = 40 from two patients; L3: *n* = 100, L5: *n* = 44, L6: *n* = 40 from three patients). These results indicate that chandelier cells mainly target the Na_V_1.6-enriched AIS region and possibly exert a strong modulatory effect on AP initiation, and thus on neuronal excitability and network activity in the human neocortex.

## DISCUSSION

Consistent with previous findings in rodents, we found no Na_V_1.1 signals at the AIS of PCs, but a spatial segregation of Na^+^ channel subtypes at the AIS, with Na_V_1.2 and Na_V_1.6 accumulated at the proximal and the distal regions, respectively. We also found, to the best of our knowledge, for the first time that at the nodes of Ranvier in adult human cortex, Na_V_1.2 was expressed by a subpopulation of nodes of Ranvier in adult human cortex in addition to the well-known presence of Na_V_1.6. Our results also show that PV-containing axonal arbors, presumably originating from chandelier cells, mainly innervate the Na_V_1.6-enriched region of the AIS but not the Na_V_1.2-enriched proximal AIS. These results suggest important roles of Na_V_1.2 and Na_V_1.6 in determining AP generation at the AIS and AP conduction along the myelinated axons of human cortical PCs, and a powerful control of chandelier cells over AP initiation (Inda et al., 2006).

Together with previous findings in rodents (Boiko et al., 2003; Van Wart et al., 2007; Duflocq et al., 2008; Lorincz and Nusser, 2008; Royeck et al., 2008; Hu et al., 2009), our results and those from others in the human cortex (Inda et al., 2006) revealed a conservative distribution pattern of Na^+^ channels, i.e., the high channel density at the AIS and the spatial segregation of Na^+^ channel subtypes. Functionally, the conservative distribution pattern could ensure a common physiological role of these channels in AP initiation and conduction. During evolution, Na^+^ channels are believed to originate from Ca^2+^ channels and evolved independently in vertebrate and invertebrate (Hille, 1984). In vertebrate, most genes of Na^+^ channel α subunits, including Na_V_1.1, Na_V_1.2, and Na_V_1.6, emerged in the stem reptilian ancestor of modern-day reptiles, birds, and mammals and retained homogenous among modern-day mammals (Zakon et al., 2011; Widmark et al., 2011; Zakon, 2012). It is of considerable interest to investigate the distribution of axonal Na^+^ channels in other mammals as well as in animals of different class and examine whether the distribution pattern is as conservative as the channel structure. Early work in frog retina showed strong immunosignals in axons but not cell bodies (Wollner and Catterall, 1986), suggesting the clustering distribution of Na^+^ channels at the axon is a conservative feature.

In this study, we found no detectable immunosignal with antibodies of Na_V_1.1, Na_V_1.2, and Na_V_1.6 at the somatodendritic compartments of cortical neurons, possibly due to a very low channel density at these locations. Previous studies also showed no labeling of Na^+^ channels in the somatic membrane (Wollner and Catterall, 1986; Inda et al., 2006; Lorincz and Nusser, 2008) or just a weak cytoplasmic labeling (Van Wart et al., 2007). Indeed, studies using quantitative electron-microscope immunogold technique (Lorincz and Nusser, 2010) and patch-clamp recording method (Kole et al., 2008; Hu et al., 2009) revealed a much lower channel density or smaller Na^+^ currents at the somatodendritic compartments, as compared with that in the axon. In our experiments, different fixation methods offered distinct immunostaining results (**Figure 1**). We found that a light fixative would offer robust signals of channel subtypes at the AIS and nodes of Ranvier. Different from weak fixation, antigen retrieval with pepsin treatment has been proved as a good method for immunostaining of Na_V_1.1 and Na_V_1.6 at the AIS in certain types of neurons (Lorincz and Nusser, 2008).

In the human cortex, immunosignals for Na_V_1.1 channels were not detected at PC axons (**Figures 4** and **6**) but they were observed at the AIS of interneurons, consistent with the results previously reported in rodents (Yu et al., 2006; Ogiwara et al., 2007; Lorincz and Nusser, 2008). Together, the results suggest an important role of this channel subtype in regulating the excitability of interneurons but not that of PCs. Our results are in agreement with previous findings that Na_V_1.1-encoding gene mutations had no effect on PC firing but could reduce the firing activity in interneurons and cause epileptic seizures (Baulac et al., 1999; Escayg et al., 2000; Claes et al., 2001; Meisler and Kearney, 2005; Catterall et al., 2010). In contrast, previous work in the human cortex revealed strong immunosignals of Na_V_1.1 at the soma of PCs (Whitaker et al., 2001a). The inconsistency could be attributable to differences in antibodies and fixation protocols.

Surprisingly, in the adult human cortical tissues, we found that a considerable subpopulation of nodes expressed Na_V_1.2 channels (**Figure 6**). Since similar results were found in both epileptic and non-epileptic adult patients, the presence of Na_V_1.2 in mature nodes was not a pathological change in the epileptic brain. In rodents, Na_V_1.2 channels are expressed in unmyelinated axons in the CNS during development; they will be replaced by Na_V_1.6 channels as myelination proceeds (Boiko et al., 2001; Kaplan et al., 2001). Among all Na^+^ channel antibodies used in this study, the two Na_V_1.2 antibodies were the most sensitive to the degree of tissue fixation. Even with low concentration of fixatives (0.5% PFA and 0.5% sucrose), perfusion of animals for a longer period of time (>2 min) would lead to false negative staining. This might explain why Na_V_1.2 signals were not found at the nodes of Ranvier in human cortex in previous studies. Previous study using expressing systems showed that, in comparison with Na_V_1.6, Na_V_1.2 exhibited a greater accumulative inactivation with high-frequency stimulations (Rush et al., 2005) and therefore unlikely supported high-frequency AP conduction. Because Na_V_1.2 displayed a more depolarized activation and inactivation threshold (Rush et al., 2005; Hu et al., 2009), the presence of Na_V_1.2 at the nodes might allow AP conduction at depolarized membrane potential levels. The accumulation of Na_V_1.2 channels at the axonal bifurcating locations (**Figure 6C5**) might ensure faithful AP conduction at these branching points where AP conduction is prone to be perturbed (Krnjevic and Miledi, 1959; Segev and Schneidman, 1999; Debanne et al., 2011). Due to the lack of suitable antibodies, double staining of Na_V_1.2 and Na_V_1.6 was not feasible in our experiments. It remains unknown whether the two subtypes are co-expressed in individual nodes.

Abnormalities in voltage-gated Na^+^ channels have been implicated in a number of epileptic syndromes (Baulac et al., 1999; Berkovic et al., 2004; Papale et al., 2009; Rakhade and Jensen, 2009; Meisler et al., 2010; Catterall, 2012). Na^+^ channel expression (channel protein or mRNA) may be subject to change during the development of epilepsy in animal models (Bartolomei et al., 1997; Gastaldi et al., 1998; Klein et al., 2004; Qiao et al., 2013) and in human patients (Lombardo et al., 1996; Whitaker et al., 2001a). In this study, we mainly focused on the distribution pattern of Na^+^ channel subtypes in human PCs and found that the distribution pattern in cortical tissues from epileptic patients was similar to that of non-epileptic patients. However, we could not exclude the possibility that Na^+^ channel density in PCs and distribution pattern in different interneuron types were subject to change with the progression of epilepsy. Moreover, our immunostaining experiments only examined the distribution patterns of Na_V_1.1, Na_V_1.2, and Na_V_1.6; we therefore could not exclude the possibility that the PC axon may express other channel subtypes, such as Na_V_1.3 and Na_V_1.5, that have been reported to be expressed in the mature cortex (Hartmann et al., 1999; Whitaker et al., 2001a; Wu et al., 2002). Whether these channel subtypes are expressed in human PC axons remains to be further examined.

Previous studies in rodents demonstrated that axo-axonic cells innervated the distal regions of the AIS of PCs (Inda et al., 2009; Wang and Sun, 2012; Inan et al., 2013; Taniguchi et al., 2013). In the human cortex, chandelier cells selectively target the distal AIS regions where K^+^ channel subtype K_V_1.2 accumulates, suggesting important roles of chandelier cells in controlling the activity of PCs (Inda et al., 2006). In this study, we further revealed that axon terminals of chandelier cells selectively targeted to AIS regions where the low-threshold Na_V_1.6 channels accumulated. The sign of postsynaptic responses (depolarization or hyperpolarization) upon activation of chandelier cells is still controversial. Because of the voltage-dependent gating properties of Na^+^ channels, membrane potential fluctuations induced by GABA release from chandelier cells and subsequent GABA receptor activation at the AIS would change the open probability of Na_V_1.6 channels and thus control the AP initiation. Chandelier cells are normally silent but their activity will increase when the overall cortical excitation increases (Zhu et al., 2004). A decrease of activity in chandelier cells could also contribute to the generation of hippocampal sharp wave (Klausberger et al., 2003; Viney et al., 2013). These findings suggest that chandelier cells may participate in the generation of different brain states by regulating PC excitability. Furthermore, chandelier cells and their modulatory effects on AP generation could be subject to changes under disease states. Previous studies showed that the density of PV-positive cartridges decreased in human subjects with epilepsy and schizophrenia (Woo et al., 1998; DeFelipe, 1999). In contrast to the distal AIS, the proximal AIS was innervated by few PV-positive axon terminals, suggesting that chandelier cells may not affect AP backpropagation toward the soma and dendrites.

In conclusion, we revealed the molecular identity of axonal Na^+^ channels at both the AIS and nodes of Ranvier of human cortical PCs. At the AIS, both Na_V_1.2 and Na_V_1.6 channels were observed along the entire AIS, but their peak immunosignals located at the proximal and distal AIS, respectively, showing a spatial segregation. At the nodes of Ranvier, most of the nodes express Na_V_1.6; however, a considerable subpopulation of nodes expresses Na_V_1.2 in adult human cortex. Our results suggest important roles of the two channel subtypes in controlling the neuronal excitability and signal conduction at the axons of human cortical PCs.

## Conflict of Interest Statement

The authors declare that the research was conducted in the absence of any commercial or financial relationships that could be construed as a potential conflict of interest.

## References

[B1] Andersen P. (1960). Interhippocampal impulses. II. Apical dendritic activation of CAI neurons. *Acta Physiol. Scand.* 48 178–208 10.1111/j.1748-1716.1960.tb01856.x 13793336

[B2] Bartolomei F. Gastaldi M. Massacrier A. Planells R. Nicolas S. Cau P. (1997). Changes in the mRNAs encoding subtypes I, II and III sodium channel alpha subunits following kainate-induced seizures in rat brain. *J. Neurocytol.* 26 667–678 10.1023/A:1018549928277 9368880

[B3] Baulac S. Gourfinkel-An I. Picard F. Rosenberg-Bourgin M. Prud’homme J. F. Baulac M. (1999). A second locus for familial generalized epilepsy with febrile seizures plus maps to chromosome 2q21-q33. *Am. J. Hum. Genet.* 65 1078–1085 10.1086/302593 10486327 PMC1288241

[B4] Berkovic S. F. Heron S. E. Giordano L. Marini C. Guerrini R. Kaplan R. E. (2004). Benign familial neonatal-infantile seizures: characterization of a new sodium channelopathy. *Ann. Neurol.* 55 550–557 10.1002/ana.20029 15048894

[B5] Boiko T. Rasband M. N. Levinson S. R. Caldwell J. H. Mandel G. Trimmer J. S. (2001). Compact myelin dictates the differential targeting of two sodium channel isoforms in the same axon. *Neuron* 30 91–104 10.1016/S0896-6273(01)00265-3 11343647

[B6] Boiko T. Van Wart A. Caldwell J. H. Levinson S. R. Trimmer J. S. Matthews G. (2003). Functional specialization of the axon initial segment by isoform-specific sodium channel targeting. *J. Neurosci.* 23 2306–2313 12657689 10.1523/JNEUROSCI.23-06-02306.2003 PMC6742039

[B7] Caldwell J. H. Schaller K. L. Lasher R. S. Peles E. Levinson S. R. (2000). Sodium channel Na(v)1.6 is localized at nodes of ranvier, dendrites, and synapses. *Proc. Natl. Acad. Sci. U.S.A.* 97 5616–5620 10.1073/pnas.090034797 10779552 PMC25877

[B8] Catterall W. A. (2012). “Sodium channel mutations and epilepsy,” in *Jasper’s Basic Mechanisms of the Epilepsies* [Internet] 4th Edn eds Noebels J. L. Avoli M. Rogawski M. A. Olsen R. W. Delgado-Escueta A. V. (Bethesda, MD: National Center for Biotechnology Information). 22787629

[B9] Catterall W. A. Kalume F. Oakley J. C. (2010). NaV1.1 channels and epilepsy. *J. Physiol.* 588 1849–1859 10.1113/jphysiol.2010.187484 20194124 PMC2901973

[B10] Claes L. Del-Favero J. Ceulemans B. Lagae L. Van Broeckhoven C. De Jonghe P. (2001). De novo mutations in the sodium-channel gene SCN1A cause severe myoclonic epilepsy of infancy. *Am. J. Hum. Genet.* 68 1327–1332 10.1086/320609 11359211 PMC1226119

[B11] Clark B. A. Monsivais P. Branco T. London M. Hausser M. (2005). The site of action potential initiation in cerebellar Purkinje neurons. *Nat. Neurosci.* 8 137–139 10.1038/nn1390 15665877

[B12] Colbert C. M. Johnston D. (1996). Axonal action-potential initiation and Na^+^ channel densities in the soma and axon initial segment of subicular pyramidal neurons. *J. Neurosci.* 16 6676–6686 8824308 10.1523/JNEUROSCI.16-21-06676.1996 PMC6579266

[B13] Coombs J. S. Curtis D. R. Eccles J. C. (1957). The interpretation of spike potentials of motoneurones. *J. Physiol.* 139 198–231 13492209 10.1113/jphysiol.1957.sp005887 PMC1358725

[B14] Craner M. J. Lo A. C. Black J. A. Waxman S. G. (2003). Abnormal sodium channel distribution in optic nerve axons in a model of inflammatory demyelination. *Brain* 126 1552–1561 10.1093/brain/awg153 12805113

[B15] Craner M. J. Newcombe J. Black J. A. Hartle C. Cuzner M. L. Waxman S. G. (2004). Molecular changes in neurons in multiple sclerosis: altered axonal expression of Nav1.2 and Nav1.6 sodium channels and Na^+^/Ca^2+^ exchanger. *Proc. Natl. Acad. Sci. U.S.A.* 101 8168–8173 10.1073/pnas.0402765101 15148385 PMC419575

[B16] Debanne D. Campanac E. Bialowas A. Carlier E. Alcaraz G. (2011). Axon physiology. *Physiol. Rev.* 91 555–602 10.1152/physrev.00048.2009 21527732

[B17] DeFelipe J. (1999). Chandelier cells and epilepsy. *Brain* 122(Pt 10), 1807–1822 10.1093/brain/122.10.1807 10506085

[B18] DeFelipe J. Gonzalez-Albo M. C. (1998). Chandelier cell axons are immunoreactive for GAT-1 in the human neocortex. *Neuroreport* 9 467–470 10.1097/00001756-199802160-00020 9512391

[B19] DeFelipe J. Hendry S. H. Jones E. G. (1989). Visualization of chandelier cell axons by parvalbumin immunoreactivity in monkey cerebral cortex. *Proc. Natl. Acad. Sci. U.S.A.* 86 2093–2097 10.1073/pnas.86.6.2093 2648389 PMC286854

[B20] Duflocq A. Le Bras B. Bullier E. Couraud F. Davenne M. (2008). Nav1.1 is predominantly expressed in nodes of Ranvier and axon initial segments. *Mol. Cell. Neurosci.* 39 180–192 10.1016/j.mcn.2008.06.008 18621130

[B21] Eccles J. C. (1964). *The Physiology of Synapses.* Berlin: Springer. 10.1007/978-3-642-64950-9

[B22] Einheber S. Zanazzi G. Ching W. Scherer S. Milner T. A. Peles E. (1997). The axonal membrane protein Caspr, a homologue of neurexin IV, is a component of the septate-like paranodal junctions that assemble during myelination. *J. Cell Biol.* 139 1495–1506 10.1083/jcb.139.6.1495 9396755 PMC2132621

[B23] Escayg A. MacDonald B. T. Meisler M. H. Baulac S. Huberfeld G. An-Gourfinkel I. (2000). Mutations of SCN1A, encoding a neuronal sodium channel, in two families with GEFS+2. *Nat. Genet.* 24 343–345 10.1038/74159 10742094

[B24] Fairen A. Valverde F. (1980). A specialized type of neuron in the visual cortex of cat: a Golgi and electron microscope study of chandelier cells. *J. Comp. Neurol.* 194 761–779 10.1002/cne.901940405 7204642

[B25] Fatt P. (1957). Sequence of events in synaptic activation of a motoneurone. *J. Neurophysiol.* 20 61–80 13398851 10.1152/jn.1957.20.1.61

[B26] Foust A. Popovic M. Zecevic D. McCormick D. A. (2010). Action potentials initiate in the axon initial segment and propagate through axon collaterals reliably in cerebellar Purkinje neurons. *J. Neurosci.* 30 6891–6902 10.1523/JNEUROSCI.0552-10.2010 20484631 PMC2990270

[B27] Fuortes M. G. Frank K. Becker M. C. (1957). Steps in the production of motoneuron spikes. *J. Gen. Physiol.* 40 735–752 10.1085/jgp.40.5.735 13428986 PMC2147645

[B28] Gastaldi M. Robaglia-Schlupp A. Massacrier A. Planells R. Cau P. (1998). mRNA coding for voltage-gated sodium channel beta2 subunit in rat central nervous system: cellular distribution and changes following kainate-induced seizures. *Neurosci. Lett.* 249 53–56 10.1016/S0304-3940(98)00394-2 9672387

[B29] Glickfeld L. L. Roberts J. D. Somogyi P. Scanziani M. (2009). Interneurons hyperpolarize pyramidal cells along their entire somatodendritic axis. *Nat. Neurosci.* 12 21–23 10.1038/nn.2230 19029887 PMC3505023

[B30] Gonzalez-Burgos G. Krimer L. S. Povysheva N. V. Barrionuevo G. Lewis D. A. (2005). Functional properties of fast spiking interneurons and their synaptic connections with pyramidal cells in primate dorsolateral prefrontal cortex. *J. Neurophysiol.* 93 942–953 10.1152/jn.00787.2004 15385591

[B31] Hartmann H. A. Colom L. V. Sutherland M. L. Noebels J. L. (1999). Selective localization of cardiac SCN5A sodium channels in limbic regions of rat brain. *Nat. Neurosci.* 2 593–595 10.1038/10147 10404176

[B32] Hille B. (1984). *Ionic Channels of Excitable Membranes.* Sunderland, MA: Sinauer Associates

[B33] Hu W. Tian C. Li T. Yang M. Hou H. Shu Y. (2009). Distinct contributions of Na(v)1.6 and Na(v)1.2 in action potential initiation and backpropagation. *Nat. Neurosci.* 12 996–1002 10.1038/nn.2359 19633666

[B34] Inan M. Blazquez-Llorca L. Merchan-Perez A. Anderson S. A. Defelipe J. Yuste R. (2013). Dense and overlapping innervation of pyramidal neurons by chandelier cells. *J. Neurosci.* 33 1907–1914 10.1523/JNEUROSCI.4049-12.2013 23365230 PMC3711719

[B35] Inda M. C. DeFelipe J. Munoz A. (2006). Voltage-gated ion channels in the axon initial segment of human cortical pyramidal cells and their relationship with chandelier cells. *Proc. Natl. Acad. Sci. U.S.A.* 103 2920–2925 10.1073/pnas.0511197103 16473933 PMC1413846

[B36] Inda M. C. DeFelipe J. Munoz A. (2009). Morphology and distribution of chandelier cell axon terminals in the mouse cerebral cortex and claustroamygdaloid complex. *Cereb. Cortex* 19 41–54 10.1093/cercor/bhn057 18440949

[B37] Kaplan M. R. Cho M. H. Ullian E. M. Isom L. L. Levinson S. R. Barres B. A. (2001). Differential control of clustering of the sodium channels Na(v)1.2 and Na(v)1.6 at developing CNS nodes of Ranvier. *Neuron* 30 105–119 10.1016/S0896-6273(01)00266-5 11343648

[B38] Kawaguchi Y. (1995). Physiological subgroups of nonpyramidal cells with specific morphological characteristics in layer II/III of rat frontal cortex. *J. Neurosci.* 15 2638–2655 7722619 10.1523/JNEUROSCI.15-04-02638.1995 PMC6577784

[B39] Khirug S. Yamada J. Afzalov R. Voipio J. Khiroug L. Kaila K. (2008). GABAergic depolarization of the axon initial segment in cortical principal neurons is caused by the Na-K-2Cl cotransporter NKCC1. *J. Neurosci.* 28 4635–4639 10.1523/JNEUROSCI.0908-08.2008 18448640 PMC6670448

[B40] Klausberger T. Magill P. J. Marton L. F. Roberts J. D. Cobden P. M. Buzsaki G. (2003). Brain-state- and cell-type-specific firing of hippocampal interneurons in vivo. *Nature* 421 844–848 10.1038/nature01374 12594513

[B41] Klein J. P. Khera D. S. Nersesyan H. Kimchi E. Y. Waxman S. G. Blumenfeld H. (2004). Dysregulation of sodium channel expression in cortical neurons in a rodent model of absence epilepsy. *Brain Res.* 1000 102–109 10.1016/j.brainres.2003.11.051 15053958

[B42] Kole M. H. Ilschner S. U. Kampa B. M. Williams S. R. Ruben P. C. Stuart G. J. (2008). Action potential generation requires a high sodium channel density in the axon initial segment. *Nat. Neurosci.* 11 178–186 10.1038/nn2040 18204443

[B43] Kole M. H. Stuart G. J. (2012). Signal processing in the axon initial segment. *Neuron* 73 235–247 10.1016/j.neuron.2012.01.007 22284179

[B44] Kordeli E. Lambert S. Bennett V. (1995). AnkyrinG. A new ankyrin gene with neural-specific isoforms localized at the axonal initial segment and node of Ranvier. *J. Biol. Chem.* 270 2352–2359 7836469 10.1074/jbc.270.5.2352

[B45] Krnjevic K. Miledi R. (1959). Presynaptic failure of neuromuscular propagation in rats. *J. Physiol.* 149 1–22 14412088 10.1113/jphysiol.1959.sp006321 PMC1363196

[B46] Lewis D. A. Lund J. S. (1990). Heterogeneity of chandelier neurons in monkey neocortex: corticotropin-releasing factor- and parvalbumin-immunoreactive populations. *J. Comp. Neurol.* 293 599–615 10.1002/cne.902930406 2329196

[B47] Lombardo A. J. Kuzniecky R. Powers R. E. Brown G. B. (1996). Altered brain sodium channel transcript levels in human epilepsy. *Brain Res. Mol. Brain Res.* 35 84–90 10.1016/0169-328X(95)00194-W 8717343

[B48] Lorincz A. Nusser Z. (2008). Cell-type-dependent molecular composition of the axon initial segment. *J. Neurosci.* 28 14329–14340 10.1523/JNEUROSCI.4833-08.2008 19118165 PMC2628579

[B49] Lorincz A. Nusser Z. (2010). Molecular identity of dendritic voltage-gated sodium channels. *Science* 328 906–909 10.1126/science.1187958 20466935 PMC3546315

[B50] Mainen Z. F. Joerges J. Huguenard J. R. Sejnowski T. J. (1995). A model of spike initiation in neocortical pyramidal neurons. *Neuron* 15 1427–1439 10.1016/0896-6273(95)90020-9 8845165

[B51] Meeks J. P. Mennerick S. (2007). Action potential initiation and propagation in CA3 pyramidal axons. *J. Neurophysiol.* 97 3460–3472 10.1152/jn.01288.2006 17314237

[B52] Meisler M. H. Kearney J. A. (2005). Sodium channel mutations in epilepsy and other neurological disorders. *J. Clin. Invest.* 115 2010–2017 10.1172/JCI25466 16075041 PMC1180547

[B53] Meisler M. H. O’Brien J. E. Sharkey L. M. (2010). Sodium channel gene family: epilepsy mutations, gene interactions and modifier effects. *J. Physiol.* 588 1841–1848 10.1113/jphysiol.2010.188482 20351042 PMC2901972

[B54] Menegoz M. Gaspar P. Le Bert M. Galvez T. Burgaya F. Palfrey C. (1997). Paranodin, a glycoprotein of neuronal paranodal membranes. *Neuron* 19 319–331 10.1016/S0896-6273(00)80942-3 9292722

[B55] Milojkovic B. A. Wuskell J. P. Loew L. M. Antic S. D. (2005). Initiation of sodium spikelets in basal dendrites of neocortical pyramidal neurons. *J. Membr. Biol.* 208 155–169 10.1007/s00232-005-0827-7 16645744 PMC5652330

[B56] Molnar G. Olah S. Komlosi G. Fule M. Szabadics J. Varga C. (2008). Complex events initiated by individual spikes in the human cerebral cortex. *PLoS Biol.* 6:e222 10.1371/journal.pbio.0060222 PMC2528052 18767905

[B57] Moore J. W. Stockbridge N. Westerfield M. (1983). On the site of impulse initiation in a neurone. *J. Physiol.* 336 301–311 6308224 10.1113/jphysiol.1983.sp014582 PMC1198971

[B58] Ogiwara I. Miyamoto H. Morita N. Atapour N. Mazaki E. Inoue I. (2007). Nav1.1 localizes to axons of parvalbumin-positive inhibitory interneurons: a circuit basis for epileptic seizures in mice carrying an Scn1a gene mutation. *J. Neurosci.* 27 5903–5914 10.1523/JNEUROSCI.5270-06.2007 PMC6672241 17537961

[B59] Palmer L. M. Stuart G. J. (2006). Site of action potential initiation in layer 5 pyramidal neurons. *J. Neurosci.* 26 1854–1863 10.1523/JNEUROSCI.4812-05.2006 16467534 PMC6793621

[B60] Papale L. A. Beyer B. Jones J. M. Sharkey L. M. Tufik S. Epstein M. (2009). Heterozygous mutations of the voltage-gated sodium channel SCN8A are associated with spike-wave discharges and absence epilepsy in mice. *Hum. Mol. Genet.* 18 1633–1641 10.1093/hmg/ddp081 19254928 PMC2667290

[B61] Peles E. Nativ M. Lustig M. Grumet M. Schilling J. Martinez R. (1997). Identification of a novel contactin-associated transmembrane receptor with multiple domains implicated in protein-protein interactions. *EMBO J.* 16 978–988 10.1093/emboj/16.5.978 9118959 PMC1169698

[B62] Planells-Cases R. Caprini M. Zhang J. Rockenstein E. M. Rivera R. R. Murre C. (2000). Neuronal death and perinatal lethality in voltage-gated sodium channel alpha(II)-deficient mice. *Biophys. J.* 78 2878–2891 10.1016/S0006-3495(00)76829-9 10827969 PMC1300874

[B63] Popovic M. A. Foust A. J. McCormick D. A. Zecevic D. (2011). The spatio-temporal characteristics of action potential initiation in layer 5 pyramidal neurons: a voltage imaging study. *J. Physiol.* 589 4167–4187 10.1113/jphysiol.2011.209015 21669974 PMC3180577

[B64] Qiao X. Werkman T. R. Gorter J. A. Wadman W. J. van Vliet E. A. (2013). Expression of sodium channel alpha subunits 1.1, 1.2 and 1.6 in rat hippocampus after kainic acid-induced epilepsy. *Epilepsy Res.* 106 17–28 10.1016/j.eplepsyres.2013.06.006 23886654

[B65] Rakhade S. N. Jensen F. E. (2009). Epileptogenesis in the immature brain: emerging mechanisms. *Nat. Rev. Neurol.* 5 380–391 10.1038/nrneurol.2009.80 19578345 PMC2822660

[B66] Rapp M. Yarom Y. Segev I. (1996). Modeling back propagating action potential in weakly excitable dendrites of neocortical pyramidal cells. *Proc. Natl. Acad. Sci. U.S.A.* 93 11985–11990 10.1073/pnas.93.21.11985 8876249 PMC38170

[B67] Regehr W. Kehoe J. S. Ascher P. Armstrong C. (1993). Synaptically triggered action potentials in dendrites. *Neuron* 11 145–151 10.1016/0896-6273(93)90278-Y 8338663

[B68] Royeck M. Horstmann M. T. Remy S. Reitze M. Yaari Y. Beck H. (2008). Role of axonal NaV1.6 sodium channels in action potential initiation of CA1 pyramidal neurons. *J. Neurophysiol.* 100 2361–2380 10.1152/jn.90332.2008 18650312

[B69] Rush A. M. Dib-Hajj S. D. Waxman S. G. (2005). Electrophysiological properties of two axonal sodium channels, Nav1.2 *and* Nav1.6, expressed in mouse spinal sensory neurones. *J. Physiol.* 564 803–815 10.1113/jphysiol.2005.083089 15760941 PMC1464456

[B70] Segev I. Schneidman E. (1999). Axons as computing devices: basic insights gained from models. *J. Physiol. Paris* 93 263–270 10.1016/S0928-4257(00)80055-8 10574116

[B71] Shu Y. Duque A. Yu Y. Haider B. McCormick D. A. (2007). Properties of action-potential initiation in neocortical pyramidal cells: evidence from whole cell axon recordings. *J. Neurophysiol.* 97 746–760 10.1152/jn.00922.2006 17093120

[B72] Somogyi P. (1977). A specific ‘axo-axonal’ interneuron in the visual cortex of the rat. *Brain Res.* 136 345–350 10.1016/0006-8993(77)90808-3 922488

[B73] Somogyi P. Freund T. F. Hodgson A. J. Somogyi J. Beroukas D. Chubb I. W. (1985). Identified axo-axonic cells are immunoreactive for GABA in the hippocampus and visual cortex of the cat. *Brain Res.* 332 143–149 10.1016/0006-8993(85)90397-X 3995258

[B74] Srinivasan Y. Elmer L. Davis J. Bennett V. Angelides K. (1988). Ankyrin and spectrin associate with voltage-dependent sodium channels in brain. *Nature* 333 177–180 10.1038/333177a0 2452986

[B75] Stuart G. Schiller J. Sakmann B. (1997a). Action potential initiation and propagation in rat neocortical pyramidal neurons. *J. Physiol.* 505 (Pt 3), 617–632 10.1111/j.1469-7793.1997.617ba.x 9457640 PMC1160040

[B76] Stuart G. Spruston N. Sakmann B. Hausser M. (1997b). Action potential initiation and backpropagation in neurons of the mammalian CNS. *Trends Neurosci.* 20 125–131 10.1016/S0166-2236(96)10075-8 9061867

[B77] Szabadics J. Varga C. Molnar G. Olah S. Barzo P. Tamas G. (2006). Excitatory effect of GABAergic axo-axonic cells in cortical microcircuits. *Science* 311 233–235 10.1126/science.1121325 16410524

[B78] Taniguchi H. Lu J. Huang Z. J. (2013). The spatial and temporal origin of chandelier cells in mouse neocortex. *Science* 339 70–74 10.1126/science.1227622 23180771 PMC4017638

[B79] Tukker J. J. Fuentealba P. Hartwich K. Somogyi P. Klausberger T. (2007). Cell type-specific tuning of hippocampal interneuron firing during gamma oscillations in vivo. *J. Neurosci.* 27 8184–8189 10.1523/JNEUROSCI.1685-07.2007 17670965 PMC6673067

[B80] Van Wart A. Trimmer J. S. Matthews G. (2007). Polarized distribution of ion channels within microdomains of the axon initial segment. *J. Comp. Neurol.* 500 339–352 10.1002/cne.21173 17111377

[B81] Viney T. J. Lasztoczi B. Katona L. Crump M. G. Tukker J. J. Klausberger T. (2013). Network state-dependent inhibition of identified hippocampal CA3 axo-axonic cells in vivo. *Nat. Neurosci.* 16 1802–1811 10.1038/nn.3550 24141313 PMC4471148

[B82] Wang X. Sun Q. Q. (2012). Characterization of axo-axonic synapses in the piriform cortex of Mus musculus. *J. Comp. Neurol.* 520 832–847 10.1002/cne.22792 22020781 PMC3903392

[B83] Waxman S. G. (2006). Axonal conduction and injury in multiple sclerosis: the role of sodium channels. *Nat. Rev. Neurosci.* 7 932–941 10.1038/nrn2023 17115075

[B84] Whitaker W. R. Faull R. L. Dragunow M. Mee E. W. Emson P. C. Clare J. J. (2001a). Changes in the mRNAs encoding voltage-gated sodium channel types II and III in human epileptic hippocampus. *Neuroscience* 106 275–285 10.1016/S0306-4522(01)00212-3 11566500

[B85] Whitaker W. R. Faull R. L. Waldvogel H. J. Plumpton C. J. Emson P. C. Clare J. J. (2001b). Comparative distribution of voltage-gated sodium channel proteins in human brain. *Brain Res. Mol. Brain Res.* 88 37–53 10.1016/S0169-328X(00)00289-8 11295230

[B86] Widmark J. Sundstrom G. Ocampo Daza D. Larhammar D. (2011). Differential evolution of voltage-gated sodium channels in tetrapods and teleost fishes. *Mol. Biol. Evol.* 28 859–871 10.1093/molbev/msq257 20924084

[B87] Wollner D. A. Catterall W. A. (1986). Localization of sodium channels in axon hillocks and initial segments of retinal ganglion cells. *Proc. Natl. Acad. Sci. U.S.A.* 83 8424–8428 10.1073/pnas.83.21.8424 2430289 PMC386941

[B88] Woo T. U. Whitehead R. E. Melchitzky D. S. Lewis D. A. (1998). A subclass of prefrontal gamma-aminobutyric acid axon terminals are selectively altered in schizophrenia. *Proc. Natl. Acad. Sci. U.S.A.* 95 5341–5346 10.1073/pnas.95.9.5341 9560277 PMC20262

[B89] Wu L. Nishiyama K. Hollyfield J. G. Wang Q. (2002). Localization of Nav1.5 sodium channel protein in the mouse brain. *Neuroreport* 13 2547–2551 10.1097/00001756-200212200-00033 12499865 PMC1579862

[B90] Yu F. H. Mantegazza M. Westenbroek R. E. Robbins C. A. Kalume F. Burton K. A. (2006). Reduced sodium current in GABAergic interneurons in a mouse model of severe myoclonic epilepsy in infancy. *Nat. Neurosci.* 9 1142–1149 10.1038/nn1754 16921370

[B91] Zakon H. H. (2012). Adaptive evolution of voltage-gated sodium channels: the first 800 million years. *Proc. Natl. Acad. Sci. U.S.A.* 109(Suppl. 1), 10619–10625 10.1073/pnas.1201884109 22723361 PMC3386883

[B92] Zakon H. H. Jost M. C. Lu Y. (2011). Expansion of voltage-dependent Na^+^ channel gene family in early tetrapods coincided with the emergence of terrestriality and increased brain complexity. *Mol. Biol. Evol.* 28 1415–1424 10.1093/molbev/msq325 21148285 PMC3058772

[B93] Zhou D. Lambert S. Malen P. L. Carpenter S. Boland L. M. Bennett V. (1998). AnkyrinG is required for clustering of voltage-gated Na channels at axon initial segments and for normal action potential firing. *J. Cell Biol.* 143 1295–1304 10.1083/jcb.143.5.1295 9832557 PMC2133082

[B94] Zhu Y. Stornetta R. L. Zhu J. J. (2004). Chandelier cells control excessive cortical excitation: characteristics of whisker-evoked synaptic responses of layer 2/3 nonpyramidal and pyramidal neurons. *J. Neurosci.* 24 5101–5108 10.1523/JNEUROSCI.0544-04.2004 15175379 PMC6729194

