# Supplementary material for: Molecular identity of axonal sodium channels in human cortical pyramidal cells
Source: Front Cell Neurosci. 2014 Sep 23;8:297. doi: 10.3389/fncel.2014.00297 (PMC4172021; doi:10.3389/fncel.2014.00297)
Supplement: Supplementary file 1 [file Data_Sheet_1.PDF]

Supplementary Material

Molecular identity of axonal sodium channels in human cortical pyramidal cells

Cuiping Tian[1], Kaiyan Wang[2],Wei Ke[4, 5], Hui Guo[3]and Yousheng Shu[4, 5 *]

[1] Institute of Neuroscience and State Key Laboratory of Neuroscience, Shanghai Institutes for Biological Sciences, Chinese Academy of Sciences, and University of Chinese Academy of Sciences, Shanghai, China;

[2]Department of Neurology, Huashan Hospital, Fudan University, Shanghai, China;

[3]Department of Neurosurgery, Shanghai Quyang Hospital, Tongji University, Shanghai, China；

[4] State Key Laboratory of Cognitive Neuroscience and Learning & IDG/McGovern Institute for Brain Research, Beijing Normal University, Beijing, China;

[5]Center for Collaboration and Innovation in Brain and Learning Sciences, Beijing Normal University, Beijing, China.

* Correspondence:

Dr. Yousheng Shu

Beijing Normal University

19 XinjiekouWai Street

Beijing 100875, P.R. China

yousheng@bnu.edu.cn

Supplementary Figures

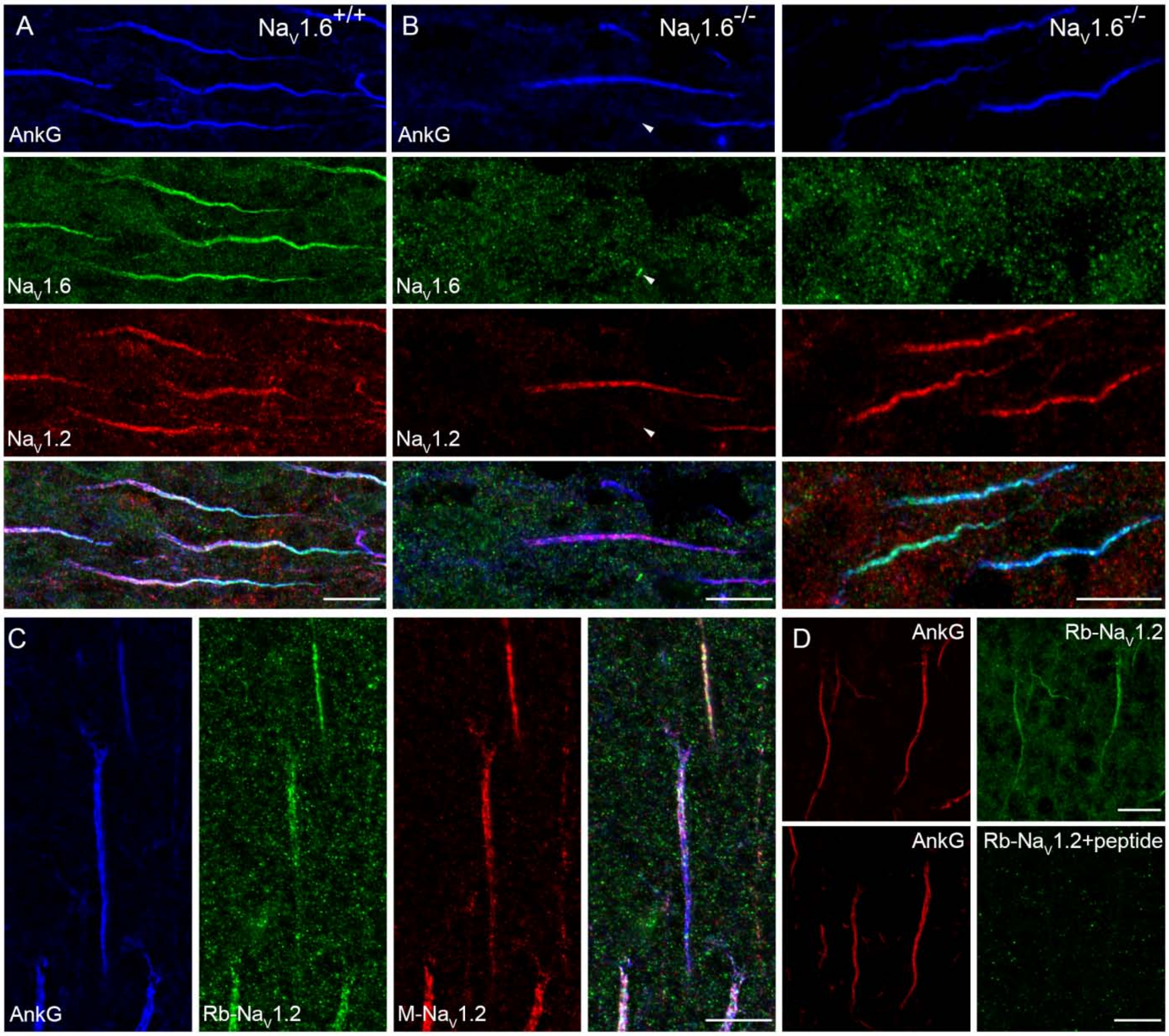

Suplementary Figures 1. Testing the specificity of $Na_V1.2$ and $Na_V1.6$ antibodies. (A，B) Triple staining of AnkG (blue), $Na_V1.6$ (green), and $Na_V1.2$ (red) in the neocortex of $Na_V1.6^{+/+}$ and $Na_V1.6^{-/-}$ mice. Note that $Na_V1.6$ immunosignals were totally absent from the AnkG-labeled AIS of the $Na_V1.6^{-/-}$ mice (B), however, robust signals were found in the control littermates (A). Immunosignals of $Na_V1.2$ were found at the AIS of all mice examined. In control siblings, they concentrated at the proximal AIS region in putative PCs, while in the $Na_V1.6^{-/-}$mice, $Na_V1.2$ immunosignals distributed relatively even along the AIS in some PCs. (B) False positive staining of $Na_V1.6$ was occasionally found in the $Na_V1.6^{-/-}$ mice (arrowhead), but showed no colabeling with AnkG. (C) Two different antibodies for $Na_V1.2$ (Rb-$Na_V1.2$ and M-$Na_V1.2$) showed similar immunoreaction profiles in the rat neocortex, indicating their high specificity. Note that $Na_V1.2$ also accumulated at the proximal portion of the AIS in the rat neocortex. (D) For the Rb-$Na_V1.2$ antibody, preabsorption with its antigen for 2 hr could completely block the staining at the AnkG-labeled AIS in the rat neocortex. The voxel depth is 3 μm in A, 2 μm and 5 μm in B, 2 μm in C, and 5 μm and 3.5 μm in D. Scale bar, 10 μm. Pia locates to the left of the section in A, B and to the top in C, D.

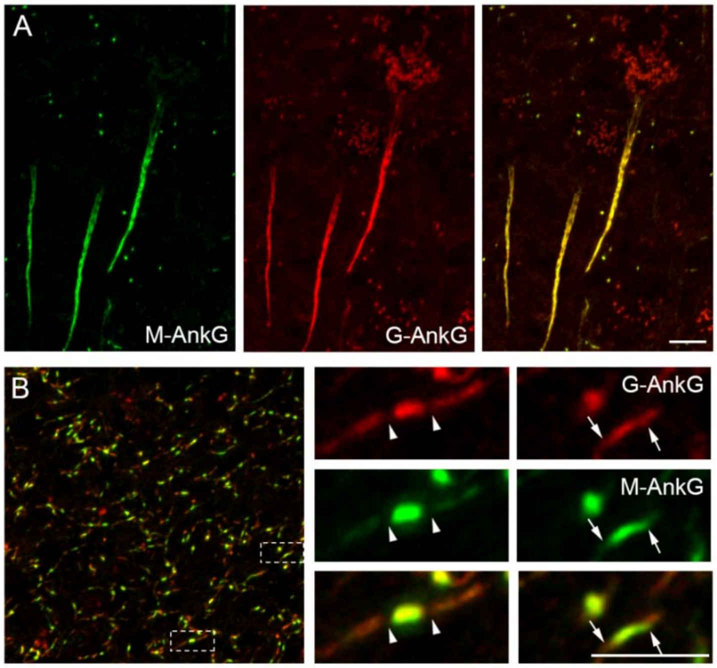

SupplementaryFigure 2. Immunostaining with AnkG antibodies at the AIS and nodes of Ranvier. (A) Immunosignals of two different AnkG antibodies generated from mouse and goat (M-AnkG, G-AnkG) overlapped at the AIS of human cortical PCs, suggesting a high specificity of these antibodies. (B) The two AnkG antibodies could also stain nodes of Ranvier in the white matter of human temporal cortex. Note that flanking regions in both sides of the node could be stained by AnkG antibodies. A gap with no detectable staining between the nodal and flanking regions could be observed (arrowheads), while in some nodes no such gap was found (arrows). The thickness of Z-stacks is 4 μm. Scale bars represent 10 μm in A, and 5 μm in B.

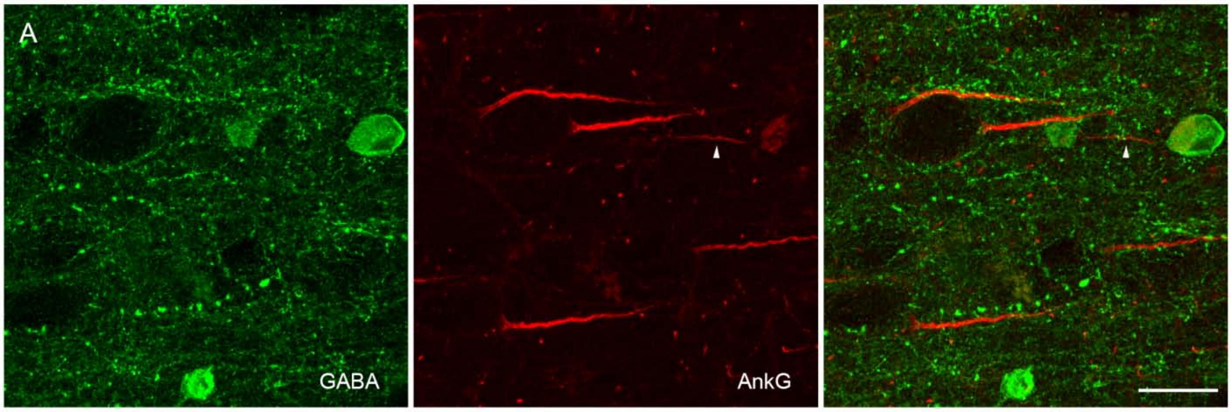

Supplementary Figure 3. Interneuron AIS is thinner than those of surrounding PCs. Double staining of GABA (green) and AnkG (red) in L3 of the anterior temporal cortex. Note that the AnkG-labeled AIS of the GABAergic neuron was much thinner than those of neighboring GABA-negative PCs. The projection direction of the interneuron AIS was different from those of PCs. The voxel depth is 6 μm and the scale bar represents 10 μm. Pia locates to the left of the section.

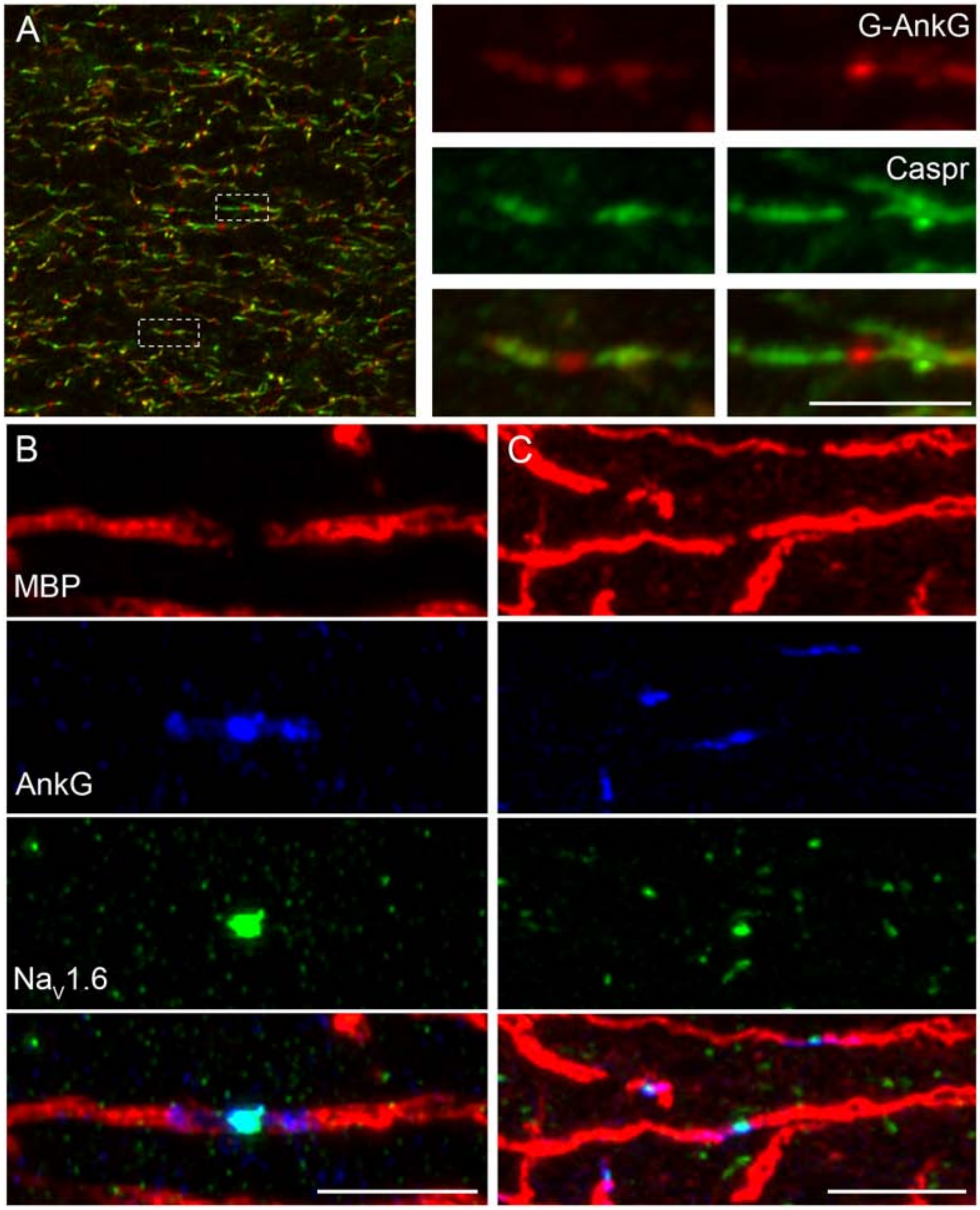

Supplementary Figure 4. Immunostaining with AnkG, Caspr and MBP antibodies at the nodes of Ranvier. (A) Double staining of Caspr (green) and AnkG (red) in the white matter of the temporal cortex. Caspr immunosignals were absent at the nodes but strong at paranodal regions. Some paranodal regions were immunoreactive to both AnkG and Caspr antibodies. (B-C) Triple staining of MBP (red), AnkG (blue) and Nav1.6 (green) in L1 of the temporal cortex. Puncta labeled for both Na$_V$1.6 and AnkG interspersed between MBP-labeled myelin sheaths. Note that MBP immunosignals located outside or directly wrapped AnkG signals at the flanking regions. The thickness of Z-stacks is 4 μm in A, 2.5 μm in B and 7 μm in C. Scale bars represent 5 μm in A and B, 10 μm in C.
